# The Transglutaminase-2 Interactome in the APP23 Mouse Model of Alzheimer’s Disease

**DOI:** 10.3390/cells11030389

**Published:** 2022-01-24

**Authors:** Micha M. M. Wilhelmus, Elisa Tonoli, Clare Coveney, David J. Boocock, Cornelis A. M. Jongenelen, John J. P. Brevé, Elisabetta A. M. Verderio, Benjamin Drukarch

**Affiliations:** 1 Department of Anatomy and Neurosciences, Amsterdam Neuroscience, Amsterdam UMC, Vrije Universiteit Amsterdam, 1081 HZ Amsterdam, The Netherlands; m.wilhelmus@amsterdamumc.nl (M.M.M.W.); k.jongenelen@amsterdamumc.nl (C.A.M.J.); jjp.breve@amsterdamumc.nl (J.J.P.B.); b.drukarch@amsterdamumc.nl (B.D.); 2 School of Science and Technology, Nottingham Trent University, Nottingham NG11 8NS, UK; elisa.tonoli@ntu.ac.uk (E.T.); clare.coveney@ntu.ac.uk (C.C.); david.boocock@ntu.ac.uk (D.J.B.); 3 Department of Biological Sciences, Alma Mater Studiorum University of Bologna, 40126 Bologna, Italy

**Keywords:** transglutaminase-2, Alzheimer’s disease, amyloid-beta, interactome, mouse model

## Abstract

Amyloid-beta (Aβ) deposition in the brain is closely linked with the development of Alzheimer’s disease (AD). Unfortunately, therapies specifically targeting Aβ deposition have failed to reach their primary clinical endpoints, emphasizing the need to broaden the search strategy for alternative targets/mechanisms. Transglutaminase-2 (TG2) catalyzes post-translational modifications, is present in AD lesions and interacts with AD-associated proteins. However, an unbiased overview of TG2 interactors is lacking in both control and AD brain. Here we aimed to identify these interactors using a crossbreed of the AD-mimicking APP23 mouse model with wild type and TG2 knock-out (TG2^−/−^) mice. We found that absence of TG2 had no (statistically) significant effect on Aβ pathology, soluble brain levels of Aβ_1–40_ and Aβ_1–42_, and mRNA levels of TG family members compared to APP23 mice at 18 months of age. Quantitative proteomics and network analysis revealed a large cluster of TG2 interactors involved in synaptic transmission/assembly and cell adhesion in the APP23 brain typical of AD. Comparative proteomics of wild type and TG2^−/−^ brains revealed a TG2-linked pathological proteome consistent with alterations in both pathways. Our data show that TG2 deletion leads to considerable network alterations consistent with a TG2 role in (dys)regulation of synaptic transmission and cell adhesion in APP23 brains.

## 1. Introduction

Alzheimer’s disease (AD) is characterized pathologically by typical lesions in the brain, in particular senile plaques (SP), neurofibrillary tangles (NFTs) and cerebral amyloid angiopathy (CAA) [1]. SP and CAA are comprised of extracellular protein aggregates enriched in multimers of amyloid-beta (Aβ) protein [2]. Aβ, in soluble and/or aggregated form, is considered as a key protein driving the disease process in AD and is therefore a major target in the development of disease-modifying (immune)therapies for AD. Unfortunately, however, at least until now, therapeutic strategies aimed specifically at reducing Aβ load suffer from inadequate efficacy, i.e., not meeting their primary clinical endpoints [3]. Different from the focus on alterations in Aβ production, accumulation and/or deposition in the brain as isolated factors in AD pathogenesis per se, the causal role of Aβ in AD has also been considered as part of a multifactorial disease process in which, in addition to Aβ, multiple other proteins are involved in key molecular and cellular mechanisms linked to neuronal dysfunction and neurodegeneration. Amongst mechanisms identified using this research strategy are neuroinflammation, oxidative stress, synaptic toxicity, cell cycle and cell membrane abnormalities and (abnormal) post-translational modification(s) of both intra- and extracellular proteins [4,5,6]. Considering the lack of success thus far with an (exclusively) Aβ-centered approach, in order to better understand AD pathophysiology and develop novel and more effective strategies for treatment, it may therefore be of utmost importance to gain more insight into such “non-amyloid factors and mechanisms”.

Transglutaminase-2 (TG2), or tissue transglutaminase, is a member of the enzyme family of transglutaminases (EC 2.3.2.13), generally known for their post-translational modification of protein substrates. TG2 is present both inside and outside of cells, including the cell surface, and has a wide range of functions, in particular crosslinking of Gln and Lys residues on protein substrates, acting as a deamidase, GTPase, isopeptidase or protein disulphide isomerase or as a molecular adapter/scaffold in non-enzymatic protein–protein interactions [7]. TG2 is involved in various (physiological and pathological) processes and conditions, including cell growth and differentiation, cell death and survival, fibrosis, inflammation and tissue repair [8]. TG2 enzymatic crosslinking activity is calcium-dependent and associated with its spatial conformation, as its compact (“closed”) conformation is generally considered as the enzymatically inactive state, whereas the stretched (“open”) conformation is associated with its enzymatic active (crosslinking) state [7]. Depending on the cellular (patho)physiology and tissue condition at hand, both TG2 spatial conformation and (sub)cellular location changes, affecting TG2 binding partners and/or enzymatic substrates with which it interacts both intracellularly and extracellularly. Therefore, this condition at hand also dictates TG2 involvement in the various cellular pathways to which its binding partners belong.

TG2 is ubiquitously expressed in neurons, glial cells and in parenchymal vessels and capillaries in the human brain [9,10]. Both TG2 enzyme as well as its crosslinking activity are elevated in AD post mortem brain tissue and cerebrospinal fluid compared to controls [11,12,13]. In addition, TG2 and its crosslinking activity are present in SPs and CAA in post mortem tissue of AD cases and in glial cells associated with these lesions [10,14]. Apart from TG2 association with AD brain lesions, it is known to directly interact and post-translationally modify soluble Aβ monomers inducing neurotoxic protein multimers [15,16,17,18,19,20]. Interestingly, TG2 also interacts with various other proteins strongly associated with the pathophysiology of AD, such as Apolipoprotein E, heparan sulphate proteoglycans, heat shock proteins, gelsolin, various mitochondrial proteins and proteins of the ubiquitin system [21,22,23,24,25]. However, an integrated view of the “protein interactome” of TG2 in AD is currently lacking. More importantly, analysis of such a TG2 interactome reveals the cellular pathways in which TG2 is involved, and how AD conditions affect TG2 involvement in pathways compared to control conditions. As a first step towards this end, in the present study we used the extensively characterized APP23 AD mimicking mouse model.

In previous work, we demonstrated the presence of TG2 and its in vivo activity in Aβ pathology and lesion-associated brain cells in APP23 mice [26]. The APP23 mouse AD model demonstrates a variety of characteristic Aβ pathologies, i.e., vascular amyloid deposits and parenchymal Aβ deposits, divided into senile plaques, small dense plaques and large diffuse anti-Aβ antibody immunoreactive areas, that develop between 12 and 24 months of age [27].

In order to analyze all TG2 interactors under both normal and AD-related disease condition to unravel the cellular pathways in which TG2 is involved, we performed a comprehensive and unbiased analysis of the proteome and TG2 interactome of APP23 and wild type (WT) animals. To establish such a comparative TG2 proteome and perform network and pathway analysis of APP23 and WT mice, crossbred animals of the APP23 mice and WT with TG2^−/−^ mice [28] were developed to exclude non-specific TG2 interactors. As TG2 is associated with both SP and its precursor diffuse plaques in AD, as well as both early vascular Aβ deposition and CAA [10,22,29], we analyzed animal brains at a disease duration (18 months) in which these Aβ pathologies are prominently present in APP23 mice, but have not yet developed to end stage disease [27]. Using this material also distribution and levels of Aβ pathology, mRNA of TG2 family members and soluble Aβ levels were determined in APP23, APP23/TG2^−/−^, WT and WT/TG2^−/−^ animals.

## 2. Materials and Methods

### 2.1. Animals

APP23 mice, overexpressing human APP751 carrying the Swedish double mutation (K670M/N671L) [27], were obtained from Novartis (generous gift from Dr. Derya R. Shimshek, Novartis Institutes of BioMedical Research, Neuroscience, Basel, Switzerland). TG2^−/−^ mice were a generous gift from Prof. Gerry Melino, and generated by deletion of 1,200 base pairs, from exon 5 to intron 6, which includes exon 6 containing the active site of TG2 [28]. C57BL/6 mice wild type (WT) were purchased from Charles River (Leiden, The Netherlands). All mice were bred within our facilities on a C57Bl/6 J background and group-housed in standard mouse cages under conventional laboratory conditions with a 12:12 h light-dark cycle (light on at 8:00 AM, light off at 8:00 PM), constant room temperature (22 ± 2 °C), humidity level (55 ± 5%), and food and water available ad libitum. Based on established milestones in the progression of AD pathology within the model (e.g., first appearance of plaques, cognitive deficits and progression of wide-spread Aβ pathology), 18-month-old mice were selected for the study [30], consisting of APP23 (*n* = 8), WT (*n* = 6), APP23/TG2^−/−^ (*n* = 10) and WT/TG2^−/−^ (*n* = 5). The experimental procedure using the above-mentioned mice were carried out in accordance with the animal welfare body of the VU University and approved by the local Animal Care and Use Committee.

### 2.2. Tissue Collection

Animals were euthanized at 18 months of age by cervical dislocation. The brains were harvested and dissected on ice into three parts: two hemi-forebrains and the cerebellum (the olfactory bulbs were discarded). After dissection, the brains were snap frozen in liquid nitrogen and immediately stored at −80 °C until use.

### 2.3. Immunohistochemistry and Double (Immuno)Fluorescence Staining

Serial coronal sections of 6 μm were obtained, starting at the base of the hippocampus. The acquired sections were fixated for 10 min using 100% acetone, unless stated otherwise. Non-specific sites were blocked using bovine serum albumin (Capricorn Scientific, Ebsdorfergrund, Germany), except for the Aβ staining for which the sections were treated with milk powder. Endogenous peroxidases were quenched using a 0.3% H_2_O_2_, 0.1% sodium azide solution in Tris-buffered saline (TBS, pH 7.6), for 15 min. All sections were incubated with their primary antibodies overnight at 4 degrees Celsius. Primary antibodies were diluted in a TBS-triton (0.5% tritonX) solution. Further details are provided in Table S1. Between the different incubation steps, sections were washed with TBS. The sections were stained for Aβ using a rabbit anti-human anti-amyloid antibody (715800, dilution 1/400) purchased from Invitrogen (Carlsbad, CA, USA). Secondary biotinylated antibody, goat anti-rabbit, was obtained from Jackson Immunoresearch (West Grove, PA, USA) and used in a 1/400 dilution. The complex of antibodies was recognized by the avidin-biotin-peroxidase complex, using the Vectastain Elite Avidin Biotin kit (Vector Laboratories, Burlingame, CA), for a period of one hour. This was done in combination with 3,3′-Diaminobenzidine (DAB) as chromogen (Sigma, St. Louis, MO, USA). After the precipitation of DAB, sections were rinsed with Tris-HCl and subsequently washed with tap water before being dehydrated in a series of alcohol dilutions, after which the sections were covered in xylene and mounted with Entellan (Merck Millipore, Darmstadt, Germany). Counterstaining was performed using hematoxylin. After counterstaining, the sections were washed with tap water once more, before being dehydrated in a series of alcohol dilutions, after which the sections were covered in xylene and mounted with Entellan (Merck Millipore).

### 2.4. Quantification of Immunohistochemical Stainings

**Figure 1:**
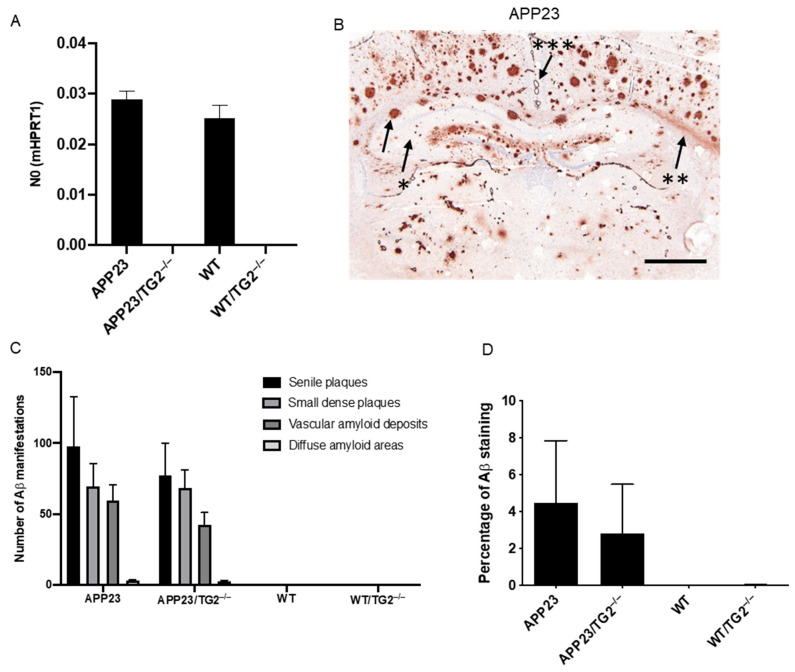
Distribution and quantification of Aβ deposits in APP23, APP23/TG2^−/−^, WT and WT/TG2^−/−^ mice brain. (**A**) TGM2 mRNA levels were determined in brain homogenates of APP23, APP23/TG2^−/−^, WT and WT/TG2^−/−^ mice. In APP23 (*n* = 9) and WT (*n* = 6) mice, TGM2 mRNA was observed, whereas TGM2 mRNA was absent in WT/TG2^−/−^ (*n* = 3) and APP23/TG2^−/−^ (*n* = 9) mice. (**B**) Analysis of Aβ deposits in post mortem cryo-fixed brain tissue of APP23 mice demonstrated different types of Aβ deposits, i.e., senile plaques (arrow), small dense plaques (arrow, asterisk), large diffuse anti-Aβ antibody immunoreactive areas (arrow, double asterisk) and vascular amyloid deposits (arrow, triple asterisk). (**C**) The number of Aβ lesions was quantified in whole brain sections. No anti-Aβ antibody immunoreactive deposits were found in WT or WT/TG2^−/−^ mice. No significant difference in the number of various anti-Aβ antibody immunoreactive deposits between APP23 and APP23/TG2^−/−^ mice was observed. (**D**) The percentage of anti-Aβ antibody immunoreactivity was analyzed as a percentage of total brain surface area. In both WT and WT/TG2^−/−^ mice, Aβ deposits were absent. No significant difference was found as a percentage of anti-Aβ antibody immunoreactivity in brain surface area between APP23 and APP23/TG2^−/−^. Scale bar: (**B**) 60 μm. Standard error of the mean is shown. Abbreviations: TG2 = transglutaminase-2, WT = wild type, Aβ = amyloid-beta, TGM2 = transglutaminase-2 coding gene.

Using a Leica brightfield microscope (DM5000B, Leica Microsystems, Wetzlar, Germany) equipped with a nuance spectral imager (Nuance 3.02, Perkin Elmer Inc., Hopkinton, MA, USA) two serial Aβ stainings were photographed per cohort, with an average of 30 μm (a minimum of 18 μm) spacing between sections of the same animal. The quantification of the Aβ burden was accomplished by performing a surface measurement of Aβ carried out by capturing a section in 4 photos at a magnification of 2.5×, following the protocol by Hepp et al. [31]. The load of Aβ pathology was represented by the percentage of the section that is covered by Aβ. In order to determine what fraction of the surface area in a section is overlaid with Aβ pathology, the multispectral imaging system used the individual spectra of DAB and hematoxylin. After discriminating between the spectra of DAB and hematoxylin the nuance software, using the co-localization tool, was able to compute the fraction of Aβ represented by DAB, that co-localizes with the hematoxylin background. To distinguish which threshold should be implemented, a test was performed with multiple sections containing a wide variety of Aβ pathology. The threshold was set at 0.200 for hematoxylin and 0.938 for Aβ, respectively. After calculating the percentage of Aβ pathology, the values of two slides per mouse were averaged, representing the amyloid-beta burden per mouse. In addition to quantification of Aβ pathology, a morphological quantification was performed to evaluate the differences between APP23 and APP23/TG2^−/−^ mice in the amount of individual amyloid manifestations. In order to do so, the protein deposits were divided into 3 morphology-based categories: senile plaques, small dense plaques and vascular amyloid deposits, respectively (see Figure 1B). Senile plaques are defined as parenchymal Aβ deposits of approximately 20–60 μm in diameter, whereas small dense plaques are approximately 2–10 μm in diameter. Vascular amyloid deposits are defined as Aβ deposits in the brain vasculature. The morphological quantification was performed by manually appointing the individual forms of Aβ deposits to one of the categories and counting these at a magnification of 4× using an Olympus Brightfield microscope (Vanox-T, Olympus Life Science Solutions, Shinjuku, Tokyo, Japan).

### 2.5. Semi-Quantitative RT-PCR

To determine the mRNA transcript levels in all mice, brain tissue was homogenized in Trizol reagent (Invitrogen, Carlsbad, CA, USA). Total RNA was isolated and 1 μg of cDNA was synthesized using the Reverse Transcription System (Promega, Madison, WI, USA) with oligo-dT primers and AMV enzyme according to the manufacturer’s instructions. For semi-quantitative RT-PCR, the SYBR Green PCR Core reagents kit (Applied Biosystems, Foster City, CA, USA) was used. Amplification of cDNA was performed in MicroAmp Optical 96-well Reaction Plates (Applied Biosystems) on an ABI PRISM 7700 Sequence Detection System (Applied Biosystems). The reaction mixture (20 μL) was composed of 1× SYBR Green buffer, 3 mM MgCl2, 875 μM dNTP mix with dUTP, 0.3 U AmpliTaq gold, 0.12 U Amperase UNG, 12.5 ng cDNA and 15 pmol of each primer (Table S2). The reaction conditions were an initial 2 min at 50 °C, followed by 10 min at 95 °C and 40 cycles of 15 s at 95 °C and 1 min at 59 °C. The mRNA expression levels were quantified relatively to the level of the housekeeping gene glyceraldehyde-3-phosphate-dehydrogenase (GAPDH) using the following calculation: 2^−(Threshold cycle of target mRNA “–’’ Threshold cycle of GAPDH)^ × 100.

### 2.6. Aβ_1–40_ and Aβ_1–42_ Protein Analysis

The concentration of Aβ_1–x_, Aβ_1–40_, and Aβ_1–42_ in the soluble protein fraction was determined by ELISA measurements using the human Aβ_1–x_ assay kit, the human Aβ_1–40_ assay kit, and the human Aβ_1–42_ assay kit (IBL International, Hamburg, Germany). All samples were diluted to within the detection limits of the test and analyzed in duplicate according to the manufacturer’s instructions. The Aβ_1–x_ assay detects all Aβ variants with an intact N-terminus and a length of more than 16 amino acids. The Aβ_1–40_ assay shows ≤0.1% cross-reactivity with other human Aβ species, but does show 16.3% cross-reactivity with endogenous Aβ_1–40_. The Aβ_1–42_ assay shows ≤0.1% cross-reactivity with other human Aβ species and endogenous Aβ (manufacturer’s instructions).

### 2.7. Brain Proteome

Five mice brains per animal group (WT, WT/TG2^−/−^, APP23 and APP23/TG2^−/−^) were weighted and homogenized in 10% *w*/*v* IP lysis buffer (25 mM Tris pH 7.4, 150 mM NaCl, 1 mM EDTA, 1% NP40, 5% glycerol; Sigma protease inhibitors) (Merck Life Science UK Limited, Gillingham, United Kingdom) using a glass-teflon Dounce homogenizer. The five APP23 and APP23/TG2^−/−^ mice were selected based on the presence of average levels of Aβ pathology when compared to the whole group. The tissue lysates were centrifuged at 13,000× *g* at 4 °C for 10 min and the supernatants used for further analysis (total brain homogenates). Equal amounts of total protein extracts (50 μg) were acetone precipitated (−80 °C overnight), followed by centrifugation at 16,000× *g* for 10 min at 4 °C and solubilization in 50 mM tri-ethyl ammonium bicarbonate (TEAB, Sigma) containing 0.1% (*w*/*v*) ProteaseMAX™ Surfactant (Promega UK, Southampton, United Kingdom). Proteins were subjected to reduction (5 mM dithiothreitol at 56 °C for 20 min), alkylation (15 mM iodoacetamide at room temperature for 15 min), and then trypsin digested overnight at 37 °C with 0.01 mg/mL MS-grade trypsin (Promega) and 0.01% (*w*/*v*) ProteaseMAX surfactant in a water bath. Samples were vacuum concentrated to dryness and resuspended in 30 µL of 5% (*v*/*v*) acetonitrile/0.1% (*v*/*v*) formic acid for MS analysis. Peptides were analyzed by RP-HPLC-ESI-MS/MS using a TripleTOF 6600+ mass spectrometer (SCIEX, Ontario, Canada). Analysis of differentially expressed proteins was performed using the OneOmics cloud processing online platform (SCIEX) as the ratio of protein peak area in APP23/TG2^−/−^ or WT/TG2^−/−^ mice over the protein peak area of the same protein in APP23 or WT mice, respectively. Data were regarded as differentially expressed at 0.545 (55%) confidence level.

### 2.8. TG2 Immunoprecipitation from Brain Homogenates

TG2 with associated proteins was immunoprecipitated from the total brain homogenate fractions using the Pierce crosslink magnetic IP kit (Fisher Scientific, Loughborough, United Kingdom) by protein A/G magnetic beads to which anti-TG2 antibody (IA12; University of Sheffield) [32] was crosslinked using disuccinimidyl suberate. Incubations of brain homogenates with the antibody-coated beads were performed for 22 h at 4 °C in constant rotation. TG2-associated proteins were subjected to reduction, alkylation and trypsin digestion directly on the beads after washing the beads three times with 50 mM TEAB. Beads were incubated for 15 h with 0.02 mg/mL of proteomics-grade trypsin (Promega) in 50 mM TEAB. Peptides were analyzed by RP-HPLC-ESI-MS/MS using a TripleTOF 6600+ mass spectrometer (SCIEX). Proteins were considered specifically associated with TG2 in WT and APP23 mice according to z-test analysis, using TG2^−/−^ cohorts as background controls, as previously described [24].

### 2.9. Information Dependent Acquisition (IDA) and SWATH Acquisition MS of Brain Homogenates and TG2 Immunoprecipitates

Brain homogenates and TG2 immunoprecipitates were analyzed by RP-HPLC-ESI-MS/MS using a TripleTOF 6600+ mass spectrometer as outlined before [24], with some modification in the protocol. The mass spectrometer was used in two different modalities depending on the stage of the experiment: information dependent acquisition (IDA) mode was employed at the beginning for spectral library construction, while SWATH 2.0-data independent acquisition (DIA) mode was used for the quantitation [33]. RP-HPLC mobile phases were solvent A (0.1% (*v*/*v*) formic acid in LC/MS grade water) and B (LC/MS grade acetonitrile containing 0.1% (*v*/*v*) formic acid). Samples were injected (trap/elute via 5 × 0.3 mm YMC Triart C_18_ trap column) onto a YMC Triart-C_18_ column (15 cm, 3 μm, 300 μm i.d) at 5 μL/min using a microflow LC system (Eksigent ekspert nano LC 425) with an increasing linear gradient of B going from 3% to 30% in 68 min, to 40% at 73 min then washing to 80% for 3 min before re-equilibration in a total time of 87 min (spectral library production by IDA), or 3% to 30% over 38 min to 40% at 43 min followed by wash, to 80% for 3 min and re-equilibrated for a total run time of 57 min (SWATH-DIA). Mass calibration (TOF-MS and Product ion) was performed every 4 samples using an injection of a standard of 40 fmol PepCal mix (SCIEX). Ionization was via the SCIEX DuoSpray™ source, using a 50 μm electrode at +5500 V. A spectral library was produced from IDA acquisitions of all samples. IDA acquisition files were searched using ProteinPilot 5.0.2 (SCIEX) and the analysis was conducted by the software with an exhaustive identification strategy, searching the Swiss-Prot database (January 2019 release, 16 January 2019) for murine species. The generated file was imported into PeakView 2.1 software (SCIEX) as an ion library and aligned to the SWATH data using endogenous peptides and exported as a .txt file after filtering for false discovery rate (FDR) of 1% and excluding shared peptides. All samples were injected again in SWATH acquisition mode using 100 variable SWATH acquisition windows with an accumulation time of 25 ms between 100–1500 *m/z* along with a single TOFMS survey scan for 50 ms between 400–1250 *m/z*, for a cycle time of 2.6 s. SWATH extraction was carried out in OneOmics (SCIEX) with the following parameters: extraction window of 5 min, maximum 30 peptides/protein, maximum 6 transitions/peptide, exclude shared peptides, and XIC width set at 30 ppm. Fold change analysis was also carried out using OneOmics. The mass spectrometry proteomics data were deposited to the ProteomeXchange Consortium via the PRIDE partner repository with the dataset identifier “PXD030354”.

### 2.10. Z-Test Statistical Analysis

The significance of protein association with TG2 was determined by *z*-test analysis [34] in the five SWATH-DIA (5 animals per group) performed on TG2-IP, using the TG2-null mice as background control. First, the protein peak area of every detected protein was normalized within the whole experiment using a *Z*-transformation: each intensity value was transformed using the natural log transformation and then normalized by subtracting the average of the entire population and dividing for the standard deviation of the entire population, as we previously described [24]. Δ*Z* values were then calculated by subtracting TG2^−/−^ *Z*-score from TG2^+/+^ *Z*-score for each protein in the APP23 or WT. Results were then plotted on a normal distribution curve to obtain probability values (*p*-values). Proteins with *p*-value lower than 0.05 detected in all 5 animals per group were regarded as significantly associated with TG2, meaning that the protein can be considered a specific partner (directly or indirectly associated) for the enzyme.

### 2.11. Bioinformatic Analysis

Functional classification and enrichment analysis of proteins of interest were performed using two different bioinformatics resources: PANTHER (Protein ANalysis THrough Evolutionary Relationships) database (www.pantherdb.org, 5 February 2021) and METACORE (https://portal.genego.com, 12 June 2020). In both cases, the whole *Mus musculus* genome was employed as background list. For the enrichment analysis of molecular functions or biological processes, we employed the statistical overrepresentation tool in Panther (Fisher exact, Bonferroni correction). Known and predicted protein-protein interactions were investigated using STRING (Search Tool for the Retrieval of INteracting Genes/proteins) database v11 (http://string-db.org, 4 May 2021). The network was produced by using the default confidence level (0.4) and by removing all the unconnected proteins and the small unconnected networks.

### 2.12. Statistical Analysis

Non-parametrical statistical analyses with exact significance values were used for all group comparisons. Comparisons between the genotype groups were performed using the independent-samples Mann-Whitney *U* test. Differences between the various age groups were evaluated with the independent-samples Kruskal–Wallis test. Post hoc analysis between specific age groups was performed using the independent-samples Mann–Whitney U test with a Bonferroni correction for multiple comparisons. All statistical tests were performed using SPSS statistics software v22.0 (IBM, Amrock, NY, USA). All graphs were created using Graphpad Prism v5.03 (Graphpad, San Diego, CA, USA).

## 3. Results

### 3.1. Distribution and Quantification of Aβ Pathology in APP23 and APP23/TG2^−/−^ Mice Brain

To confirm the complete absence of TG2 mRNA in the newly developed crossbred mice, TGM2 mRNA levels were analyzed in brain homogenates of APP23, WT, APP23/TG2^−/−^ and WT/TG2^−/−^ mice. In both APP23 (*n* = 9) and WT (*n* = 6) mice, TGM2 mRNA was observed (Figure 1A). In contrast, in both WT/TG2^−/−^ (*n* = 3) and APP23/TG2^−/−^ (*n* = 9) mice, TGM2 mRNA was absent (Figure 1A). The trend increase in TGM2 in APP23 compared to WT was not significant.

In APP23 mice, initial Aβ deposits were observed at the age of 6 months and increased in both number and surface area with age [27]. In brain tissue of 12- to 24-month-old APP23 mice, Aβ deposits are abundantly present and different types of Aβ deposits have been described, i.e., vascular amyloid deposits and parenchymal Aβ deposits, divided into senile plaques, small dense plaques and large diffuse anti-Aβ antibody immunoreactive areas [27]. To analyze the effect of the absence of TG2 on Aβ deposits, immunohistochemical analysis on cryo-fixed post mortem brain tissue using an anti-human Aβ antibody was performed on both APP23 and the newly developed crossbred APP23/TG2^−/−^ mice. In both APP23 and APP23/TG2^−/−^ mice, the above-described types of Aβ pathology were observed (Figure 1B).

In order to quantify the effect of the absence of TG2 on the different type of Aβ lesions, differences in number of Aβ lesions are quantified in whole brain slices. We found no anti-Aβ antibody immunoreactive deposits in WT and WT/TG2^−/−^ mice brains (Figure 1C). Analysis of the total number of senile plaques, small dense plaques, vascular amyloid deposits and diffuse amyloid areas in APP23 and APP23/TG2^−/−^ mice showed no significant difference between the two groups (Figure 1C). To determine whether the absence of TG2 significantly affects the overall Aβ load in these mice, the percentage of anti-Aβ antibody immunoreactivity was analyzed as a percentage of total brain surface area [31]. In both WT and WT/TG2^−/−^ mice, no Aβ deposits were detected (Figure 1D). In line with our quantitative analysis of the number of individual Aβ lesions, the percentage of total brain anti-Aβ antibody immunoreactivity demonstrated no significant difference in anti-Aβ antibody immunoreactivity as a fraction of brain surface area between APP23 and APP23/TG2^−/−^ mice (Figure 1D).

### 3.2. Analysis of mRNA of Human APP, Mouse TGM1, TGM3, TGM6 and FXIIIA and Soluble Brain Aβ_1–40_ and Aβ_1–42_ Levels, and Aβ_40/42_ Ratio in Mouse Brain Homogenates

**Figure 2:**
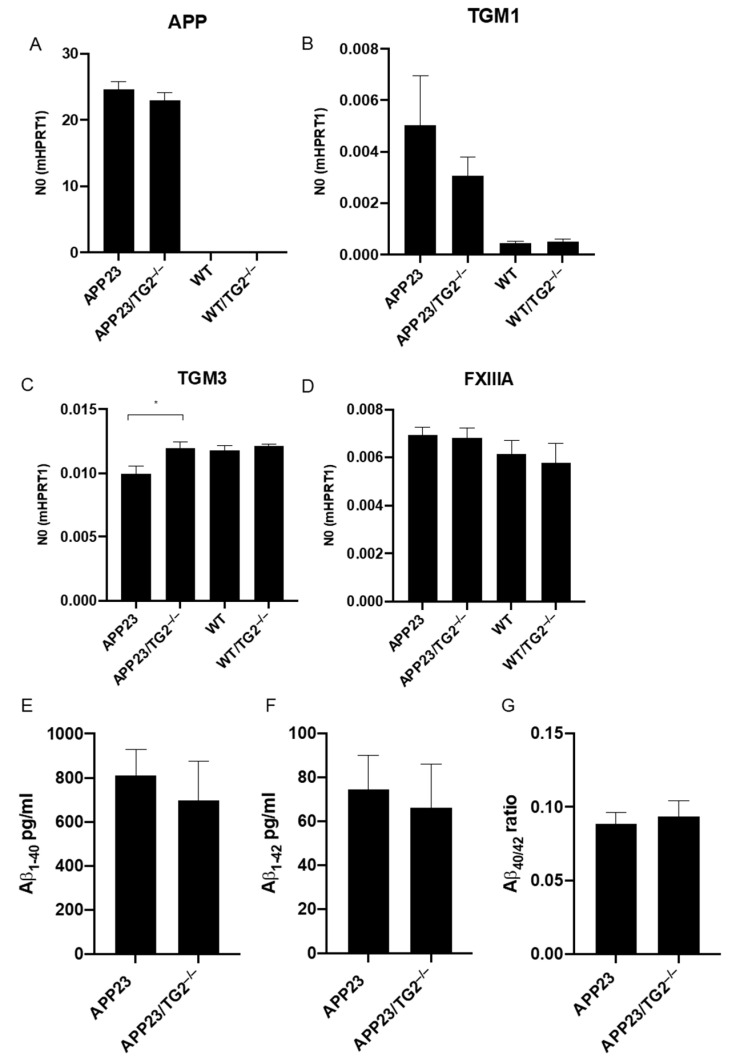
Analysis of mRNA of human APP, mouse TGM1, TGM3, TGM6 and FXIIIA, and soluble brain Aβ_1–40_ and Aβ_1–42_ levels, and Aβ_40/42_ ratio in mouse brain homogenates. (**A**–**D**) Levels of mRNA of APP, TGM1, TGM3 and FXIIIA were analyzed in mouse brain homogenates of WT, WT/TG2^−/−^ APP23 and APP23/TG2^−/−^. (**A**) No significant difference in human APP mRNA levels were found between APP23 and APP23/TG2^−/−^ mice. No human APP mRNA was observed in both WT and TG2^−/−^ mice. No significant increase in mRNA levels between APP23 or WT mice and APP23/TG2^−/−^ and TG2^−/−^, respectively, for TGM1 (**B**), TGM3 (**C**), and FXIIIA (**D**) was found. For TGM3, a significant increase (* *p* = 0.03) was observed between APP23 and APP23/TG2^−/−^ mice (**C**). (**E**–**G**) Soluble human Aβ_1–40_, Aβ_1–42_ and Aβ_1–40_/Aβ_1–42_ ratio were analyzed in mouse brain homogenates. No significant difference in both soluble brain Aβ_1–40_ and Aβ_1–42_ levels were found in APP23/TG2^−/−^ mice compared to APP23 mice (**E**,**F**). No significant difference in soluble brain Aβ_1–40_/Aβ_1–42_ ratio was found between APP23 and APP23/TG2^−/−^ mice (**G**). Standard error of the mean is shown. Abbreviations: TG2 = transglutaminase-2, TGM1 = transglutaminase-1 coding gene, TGM3 = transglutaminase-3 coding gene, APP = amyloid-beta precursor protein coding gene, FXIIIa = factor 13a, Aβ = amyloid-beta.

Absence of TG2 might result in upregulation of other TG family members [35]. In order to investigate this, mRNA levels of other TG family members known to be expressed in the human brain, i.e., TG1, TG3, TG6 and FXIIIa, were analyzed. mRNA levels in mouse brain homogenates demonstrated no significant difference in human APP mRNA levels between APP23 and APP23/TG2^−/−^ (Figure 2A). As expected, no human APP mRNA was observed in both WT and WT/TG2^−/−^ mice (Figure 2A). Analysis of TG2 family members expressed in the mouse brain demonstrated no significant increase in mRNA levels between APP23 or WT and their TG2^−/−^ counterparts for TGM1 (Figure 2B), FXIIIA (Figure 2D) and TGM6 (data not shown as TGM6 mRNA level were not significantly higher compared to background), with the exception of TGM3 mRNA levels which were different between APP23 and APP23/TG2^−/−^ mice but not between WT and WT/TG2^−/−^ (Figure 2C). This suggests that TGM3 expression is reduced in APP23 mice when compared to APP23/TG2^−/−^ mice, but also, albeit not significantly, when compared to WT and WT/TG2^−/−^ mice.

As Aβ interacts with and is a substrate of TG2 [19], we analyzed the effects of the absence of TG2 on levels of soluble brain Aβ_1–40_, Aβ_1–42_ and/or Aβ_40/42_ ratio using a dedicated ELISA. Although a reduction in both soluble brain Aβ_1–40_ and Aβ_1–42_ levels was observed in APP23/TG2^−/−^ mice compared to APP23 mice, no significant difference was found (Figure 2E,F). Analysis of Aβ_1–40_ /Aβ_1–42_ ratio also demonstrated no significant difference in soluble Aβ_1–40_ /Aβ_1–42_ ratio between APP23 and APP23/TG2^−/−^ mice (Figure 2G).

### 3.3. Quantitative Comparative Proteomics of TG2 Binding Partners

**Figure 3:**
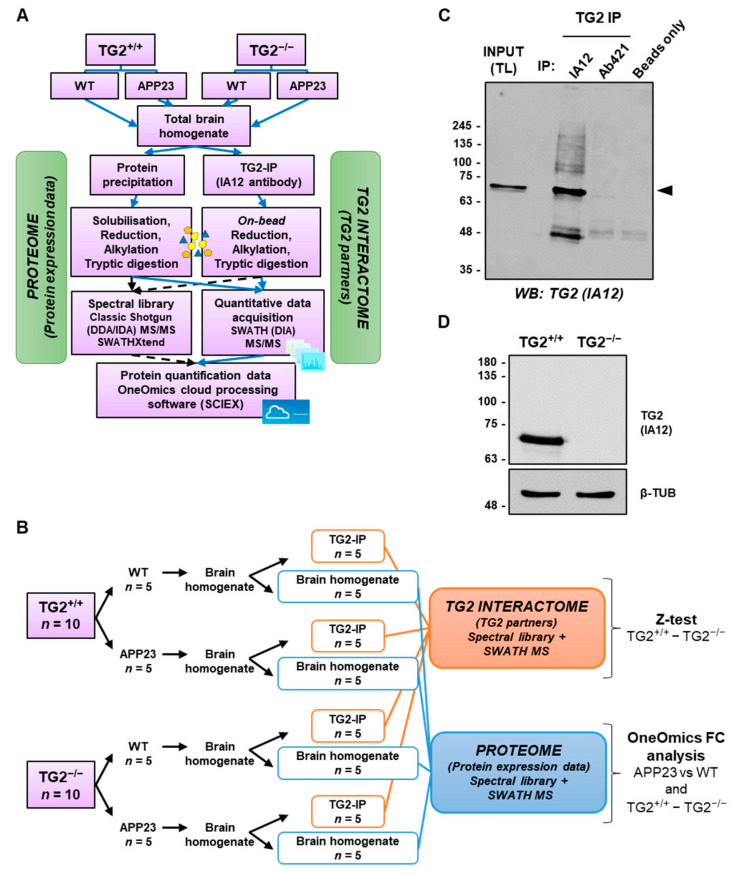
Analysis of TG2 interactome and total proteome in mouse brain by comparative proteomics. (**A**) Workflow describing the approach used for the isolation of TG2-interacting proteins. TG2 was immunoprecipitated from total homogenates obtained from WT, APP23, WT/TG2^−/−^ and APP23/TG2^−/−^ brains (with TG2^−/−^ cohorts used as negative controls) using magnetic beads crosslinked with a mouse monoclonal anti-TG2 antibody (IA12). TG2 co-immunoprecipitated proteins (TG2-IP) were trypsin digested on beads and analyzed by SWATH MS. SWATH quantitative data were extracted using a spectral library produced by shotgun/data dependent acquisition (DDA/IDA) MS on all TG2-IP samples and total brain lysates. The TG2 interactome was generated via evaluation of differences between TG2^+/+^ and TG2^−/−^ precipitated proteins (background) by using a paired sample z-test. (**B**) Sample size (*n*) used in the study. (**C**) TG2 was immunoprecipitated from a mouse brain total lysate (WT) by Pierce Crosslink Magnetic IP/Co-IP Kit as described in the Methods, using either mouse monoclonal anti TG2 (IA12) or rabbit polyclonal anti-TG2 (Ab421) antibodies. TG2-IP samples were separated by reducing SDS-PAGE (10% *w*/*v*) and subjected to Western blot for TG2 using IA12 antibody. Black triangle denotes TG2. The brain total lysate (input, TL) from a WT mouse was used as loading control (50 μg). (**D**) Total cell lysates from WT and WT/TG2^−/−^ mouse primary astrocytes were subjected to WB and probed with IA12 antibody (10 μg).

We performed quantitative proteomics by sequential window acquisition of all theoretical fragmentation spectra (SWATH) mass spectrometry (MS) on TG2-immunoprecipitated whole brain homogenates of APP23 and WT mice, using the APP23/TG2^−/−^ and WT/TG2^−/−^ as a control. This unbiased approach was used to detect protein–protein complexes ex vivo. The TG2 IP proteome from both WT/TG2^−/−^ (*n* = 5) and APP23/TG2^−/−^ (*n* = 5) was subtracted from the respective TG2 IP WT (*n* = 5) or APP23 (*n* = 5) proteome to reveal only the TG2-dependent interactions. The outline of this original approach is shown in Figure 3A,B. TG2-associated complexes were isolated by IP using magnetic beads coated with an anti-TG2 antibody (mouse monoclonal IA12) [32] which was validated for ability to immunoprecipitate mouse TG2 in comparison with another polyclonal anti-TG2 antibody (Figure 3C), and for specificity using TG2^+/+^ and TG2^−/−^ primary mouse cell lysates as negative control (Figure 3D). In order to resolve proteomes at the highest possible sensitivity, reproducibility, and proteome coverage, the above-mentioned SWATH acquisition was used. Five IP per cohort, each starting from total lysates generated from five animal donors from each mice model, as well as the total homogenates, were used to build the spectral library to avoid bias from individual donors and achieve generalizable results (Figure 3B).

**Figure 4:**
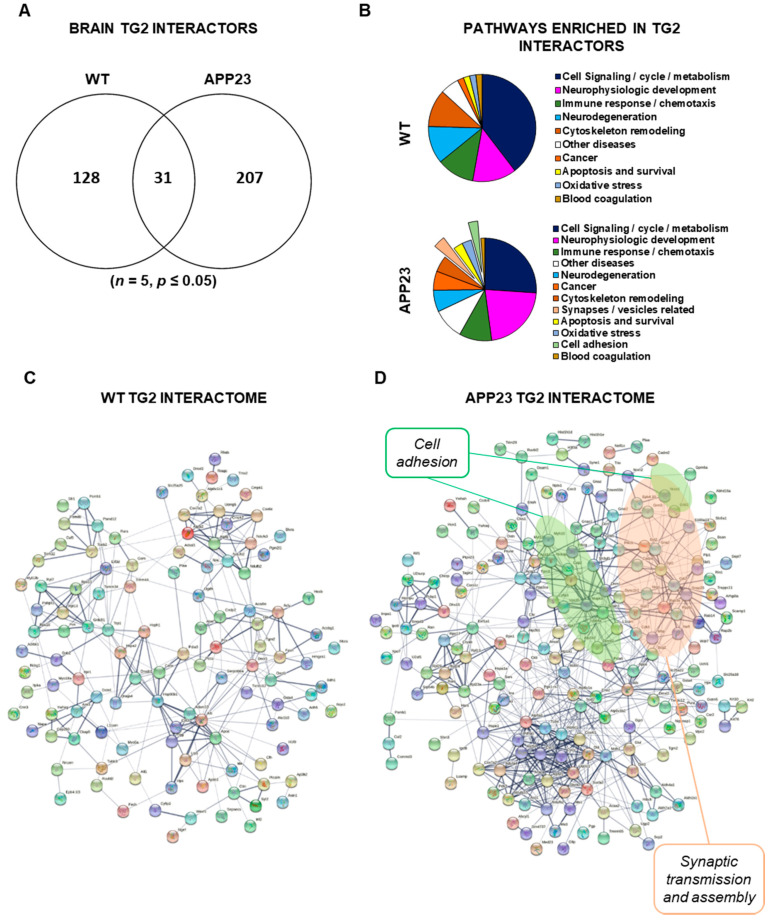
Analysis of TG2-associated proteins in brain reveals an increase in TG2 partners in APP23 animals compared to WT. (**A**) Number of proteins identified by comparative proteomics as specifically associated with TG2 in APP23 and WT brains by *z*-test (*p* ≤ 0.05; *n* = 5), using the workflow shown in Figure 3A,B. (**B**) Pie charts display the distribution of the enriched pathways of TG2-associated proteins in WT (53 enriched pathways) and APP23 (263 enriched pathways) according to METACORE “pathway maps” analysis, manually grouped in macro-categories. The area of each slice is proportional to the number of enriched pathways it comprises. TG2 partners which were in common between WT and APP23 were not included in this analysis. (**C**,**D**) The protein interaction network built from TG2-associated proteins in WT (**C**) and APP23 (**D**) was mapped against the *M. musculus* reference database using String V11.0 (http://stringdb.org, 4 May 2021). The map was built by considering both known and predicted protein interactions with the default threshold confidence level of 0.4. The thickness of the lines is proportional to the confidence of the interactions.

Analysis of the TG2 interactome (Figure 4A) highlighted a clear change in TG2 partners from WT to APP23 brain, with a 50% increase of TG2 interactors in the disease model, of which 13% were in common with the WT interactome (Figure 4A). Specifically, 159 proteins were the TG2 partners in the WT brain and 238 proteins in the APP23 brain, of which 31 proteins were in common (Figure 4A and Table S3). Analysis of pathway maps (Metacore) showed an enrichment of TG2-associated proteins in the macro-categories of cell adhesion (e.g., actin, vinculin and beta-tubulin) and synaptic vesicles-related pathways (e.g., RAB3A, synaptogamin and NPTX1) uniquely in APP23 brain (Figure 4B). Network analysis of the TG2 interactomes performed by STRING (built based on known and predicted protein–protein interactions) (Figure 4C,D) revealed new clusters of cell adhesion and synaptic vesicles-related proteome in the APP23 TG2 interactome, which were absent in the WT TG2 interactome. A protein cluster related to mitochondrial energy metabolism was identified in both networks (lower portion of the map) but this was enlarged and denser in APP23, suggesting a link of TG2 with mitochondrial stress typical of Aβ-associated pathology [36].

**Figure 5:**
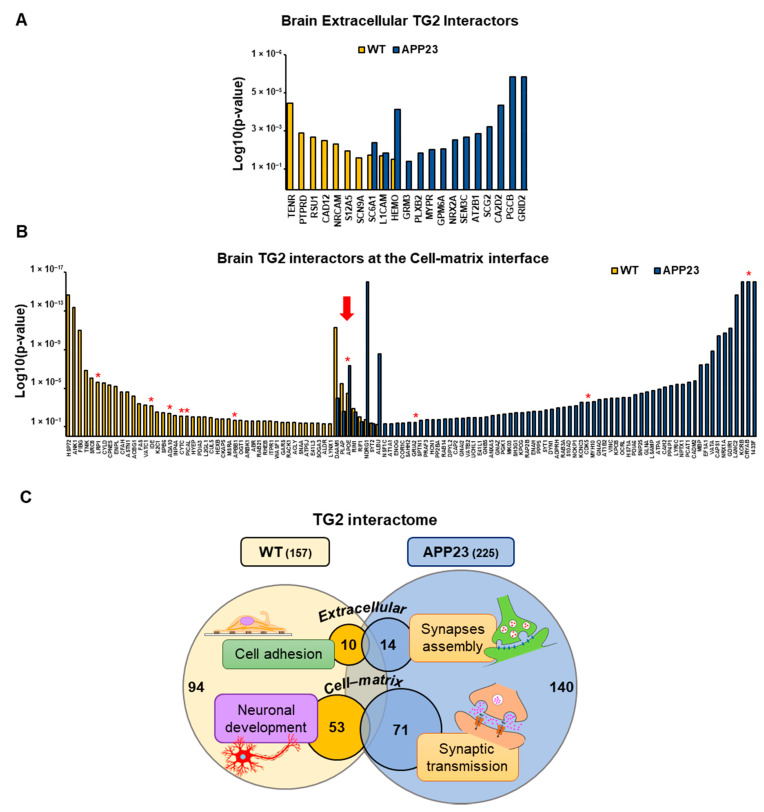
TG2-interacting proteins localized in the extracellular space and cell–matrix interface. (**A**) TG2 interactors in WT and APP23 exclusively localized at the plasma membrane and extracellular space according to PANTHER analysis (GO database Cellular Component-Complete). Proteins are listed in order of significance of their association with TG2 (Log10 *p*-value, *z*-test). (**B**) TG2 partners in WT and APP23 localized at the cell–matrix interface according to PANTHER analysis. Red asterisks indicate proteins linked to amyloid β. The red arrow indicates APOE. (**C**) Chart visualizing the TG2 interactome in WT and APP23 brain, including only proteins localized extracellularly or at the cell–matrix interface, with highlight of enriched GO molecular functions according to PANTHER analysis (GO database Molecular Function-Complete). Immunoglobulins were manually removed from these analyses.

When TG2-interacting proteins were classified according to their cellular localization (Panther), a cluster of TG2 interactors was found exclusively localized at the cell membrane and extracellular space (Figure 5A, Table 1 and Table 2) (about 11% of APP23 interactome and 8% of WT interactome) and a cluster at the cell–matrix interface (Figure 5B, Table 1 and Table 2), (about 30% of APP23 interactome and 33% of WT interactome). Among these, APOE was confirmed as a strong TG2 partner in APP23 brain (*p* = 4.3 × 10^−8^) and WT brain (*p* = 3.5 × 10^−5^) (Figure 5B, red arrow). The TG2 interactome included a series of other Aβ-interacting proteins in both WT and APP23 brain (e.g., glutamate receptor 2, cyclin-dependent-like kinase 5, insulin-degrading enzyme, disintegrin and metalloproteinase domain-containing protein 10, phosphatidylinositol-binding clathrin assembly protein, amyloid-beta A4 precursor protein-binding family B member 1; Figure 5B, red asterisks). Biological process analysis of these subgroups of TG2-interacting proteins (Panther) revealed a significant enrichment of cell adhesion and brain development functions in WT brain and of synapses assembly and synaptic transmission in APP23 brain (Figure 5C). Notably, of the 207 TG2 partners restricted to the APP23 brain (Table S3) only two of the detected proteins were slightly overexpressed in the APP23 total proteome compared to the WT proteome, as shown in the next section (Table 3), thus ruling out a concentration-dependent partnership with TG2.

Together, these data suggest that the array of TG2 interactors undergoes a clear change from WT to APP23 brain at 18 months and shifts towards synapse-related functions in the presence of Aβ pathology.

### 3.4. TG2^+/+^ and TG2^−/−^ Brain Proteomes in APP23 Mouse Model

**Figure 6:**
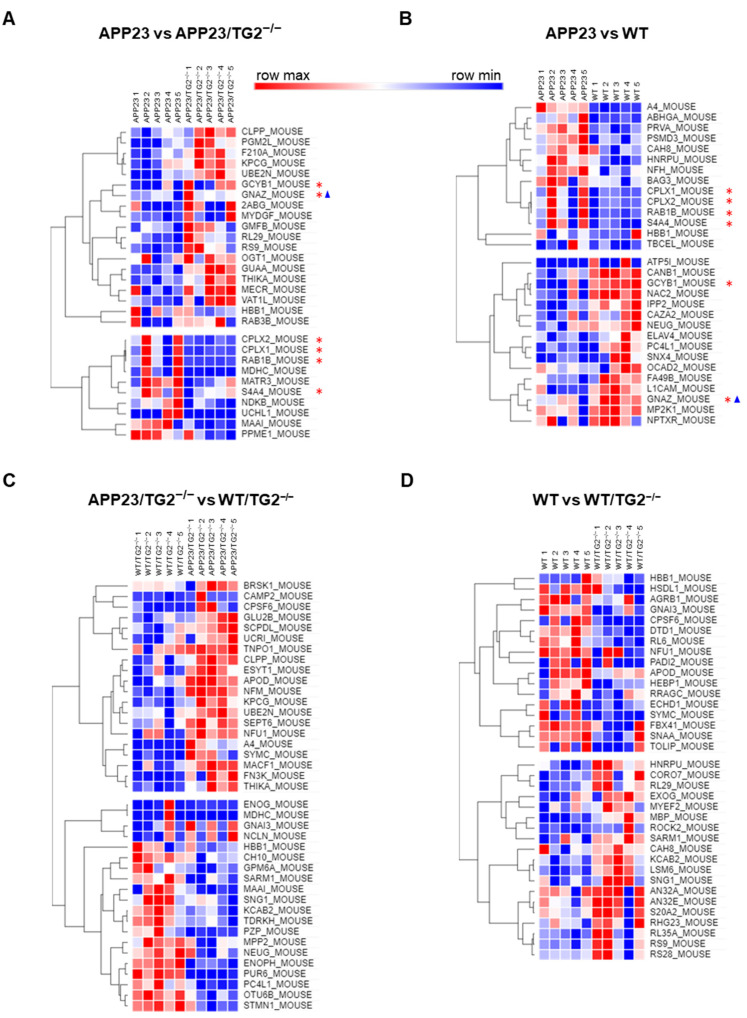
Analysis of brain proteome reveals significant changes between WT and APP23, and the effect of TG2 knock-out on protein expression. (**A**–**D**) Heat maps of each comparison (confidence ≥ 55%). The red asterisks in (**B**) indicate proteins changed in expression level in APP23 compared to WT, which are also dependent on TG2 expression (change in APP23 reversed in APP23/TG2^−/−^ proteome as shown in (**A**)). The blue delta (Δ) indicates which of these APP23-linked TG2-expression-dependent proteins are also TG2 interactors (with reference to Figure 4). Each row maximum value is depicted in red and minimum value in blue, with intermediate values indicated with shades in between.

Having identified the specific TG2-associated protein network in the APP23 brain-TG2 precipitates, we extended the analysis of the TG2-linked pathological proteome to those proteins which do not necessary physically interact with TG2, but concur to TG2-mediated AD pathology. Quantitative proteomics was employed to compare the healthy and diseased (APP23) brain. Comparison of APP23 with APP23/TG2^−/−^ brains (Figure 6A) highlighted proteins specifically linked with expression of TG2: Ras-related protein Rab-1B (RAB1B); Complexin-1 and -2 (CPLX1 and CPLX2) and Electrogenic sodium bicarbonate cotransporter 1 (S4A4) (log_2_ (APP23/APP23 TG2^−/−^) > 0, Table 4). These proteins were increased in the wild type APP23 brains and decreased in the APP23 brains lacking TG2 (Figure 6A); moreover, they were involved in the APP23 pathology as consistently increased in the APP23 brains compared to WT brains (Figure 6B) (log_2_ (APP23/WT) > 0, Table 3). Notably, CPLX2 was also found to be increased in previous proteomic analysis of the APP23 mouse model, although CPLX1 was decreased initially in 2-month-old mice [37,38].

Conversely, Guanylate cyclase soluble (sGC) subunit beta-1 (GCYB1) and G Protein Subunit Alpha Z (GNAZ) consistently decreased in the wild type APP23 brains compared to the APP23/TG2^−/−^ brains where they were more expressed (Figure 6A) (log_2_ (APP23/APP23 TG2^−/−^) < 0, Table 4) and they were involved in the APP23 pathology being decreased in APP23 brains compared to WT (Figure 6B) (log_2_ (APP23/WT) < 0, Table 3).

Comparative proteomics of the APP23/TG2^−/−^ brains versus the WT/TG2^−/−^ brains (Figure 6C, Table 5) failed to detect differences in the above-mentioned proteins between these mice cohorts, thus excluding that these proteins changed independently from TG2 expression. Furthermore, none of the TG2-related proteins altered in APP23 compared to WT identified in this study (Figure 6A,B, red asterisks in the heat maps) changed in the WT brains following TG2^−/−^ (Figure 6D, Table 6), underscoring our interpretation that they are part of a TG2-linked pathological proteome associated with the disease (APP23) phenotype.

ApoE, a well-known risk factor of AD and linked to Aβ pathology in APP23 mice in previous work [21,37,38], was revealed as a TG2 partner in both the WT and the APP23 interactome (Figure 5B, Table 2) and was also found to be increased in the APP23 brains in this study but at a lower level of confidence (54%).

## 4. Discussion

We here for the first time provide an unbiased overview of TG2 interactors and their pathways in both “normal” and an Aβ pathology-mimicking condition, using TG2^−/−^ mouse models as a control. Network analysis of the TG2 interactome revealed a 50% increase of the number of TG2 interactors in the APP23 model compared to WT, and a clear change in the cellular pathways of which these interactors are part. Interestingly, under APP23 conditions, TG2 interactors linked to synaptic vesicle trafficking and cell adhesion pathways were added to the pathways observed in the WT condition. In addition, the number of TG2 interactors which were part of a protein cluster related to mitochondrial energy metabolism was enlarged in APP23 compared to WT. Apart from the pathway analysis, cellular location analysis of TG2 interactors revealed clusters of proteins present at the cell membrane and cell–matrix interface, and biological process analysis demonstrated that TG2 interactors are involved in cell adhesion and synaptic transmission. In line with these data, comparative proteomics showed that TG2 deletion resulted in (stronger) association of TG2 with the proteins part of synaptic transmission, mitochondrial function, membrane trafficking and signaling pathways in APP23 brains compared to WT. Together, these data show a strong shift in both number and cellular function of TG2 interactors between control and disease condition, and provide novel insight into the role of TG2 in development and/or progression of Aβ pathology and related cellular processes.

In both 18-month-old APP23 and APP23/TG2^−/−^ mice, a variety of Aβ pathology, i.e., senile plaques, small dense plaques and vascular Aβ deposits was observed, as expected [27]. As 18-month-old APP23 mice are considered to be in a “moderate” state of disease progression [27], compared to end-stage disease observed in 24-month-old animals, variation in Aβ pathology and load between animals of the same group did not come as a surprise. However, given the proposed role of TG2 in Aβ development and disease progression [39], interactors of TG2 found at this stage of the disease might be more relevant as potential therapeutic targets compared to end-stage disease interactors. Interestingly, despite the accumulating evidence that TG2 plays an important role in development and progression of Aβ pathology in both AD [39] and in the APP23 mouse model [26], in the current study absence of TG2 did not lead to significant differences in Aβ load and pathology, between APP23 and APP23/TG2^−/−^ mice. In addition, no statistical differences in soluble Aβ brain levels of Aβ_1–40_, Aβ_1–42_ and Aβ_40/42_ ratio were observed between APP23 and APP23/TG2^−/−^ mice, although the levels measured are in line with previous publications and demonstrated a typical 10-fold increase between soluble Aβ_1–40_ and Aβ_1–42_ levels [40]. Furthermore, with the exception of TGM3, the absence of TG2 did not result in the increase in mRNA levels of other TG2 family members, as reported previously in a Parkinson’s disease model [35]. These data demonstrate that both animal models, i.e., APP23 and APP23/TG2^−/−^, are ideal for TG2 interactome and proteome comparison, as the absence of TG2 did not affect Aβ pathology and levels and did not give rise to compensatory alterations in expression of other TG family members.

Analysis of the TG2 proteome of APP23 and WT mice demonstrated a strong shift in TG2 interactors between mice models. Interestingly, the 159 TG2 interactors found in WT mice increased to 238 interactors in APP23 mice, with only 31 interactors in common. This demonstrated that the expression and accumulation of human Aβ in APP23 mice has a robust effect on the panel of TG2 interactors when compared to its WT counterpart. Our findings are in line with previous TG2 interactome analysis between control and disease state, i.e., a kidney fibrotic mouse model, in which a similar strong shift in TG2 interactors between control and disease state was observed [24]. This dramatic shift in interactors is most likely related to TG2 pleiotropic functions in and outside the cell [7]. Driven by the cellular state at hand, TG2 localization and conformation changes, related to its catalytically active (open) or inactive (closed) state, lead to different binding partners and/or substrates [7,41]. Of special interest are the identified TG2 binding partners unique to APP23 mice and well-known players in Aβ pathophysiology and neurodegeneration such as the glutamate receptor in the extracellular space or cell membrane compartment [42], and 14-3-3 protein and alpha-B-crystallin in the cell–matrix compartment [29,43,44]. In addition to their role in AD, both 14-3-3 and alpha-B-crystallin are known interactors and substrates of TG2, respectively [45,46]. Alike TG2, 14-3-3 protein plays a role in cell survival and the autophagy pathway and both their expression is altered in AD, suggesting a possible connection to neurodegeneration in AD [43,47]. Our findings also hint towards an Aβ-driven process in which TG2 crosslinks alpha-B-crystallin, thereby modifying Aβ-induced cytotoxicity, as suggested previously [44,45], or hampering alpha-B-crystallin physiological functioning in recognizing misfolded proteins [48]. In addition, amongst the TG2 interactors common for APP23 and WT is the well-known AD risk factor and key player in both Aβ-pathophysiology and AD-related neurodegeneration [49,50], Apolipoprotein E (ApoE), which we recently identified as a substrate for TG2-catalyzed crosslinking [21]. Interestingly, the *p*-value of ApoE decreased approximately 800-fold in APP23 mice, compared to WT, suggesting that it is more strongly associated with TG2 in disease. Although the role of ApoE in the formation of the typical AD brain lesions and neurodegeneration is still under debate, our data suggest a possible link with TG2 that might modify ApoE at the post-translational level under pathological conditions such as AD. 

In addition to separate individual interactors of TG2 observed under control and Aβ pathology conditions, pathway and network analyses of our data demonstrated a unique enrichment of TG2-associated proteins in cell adhesion- and synaptic vesicles-related pathways in APP23 mice. In AD, Aβ-dependent changes in synaptic adhesion affect the function and integrity of synapses, suggesting that alterations in synaptic adhesion play key roles in the disruption of neuronal networks, resulting in neurodegeneration [51]. TG2 is also closely linked to the cell adhesion process, in which it interacts with an array of matrix molecules such as integrin, growth factor receptors, and other cell surface or extracellular matrix proteins, in particular fibronectin and heparan sulfate proteoglycans, to trigger adhesion signaling [52,53,54]. Modifications in the synaptic vesicle-related pathways are also well known for AD, as results of human and animal AD model studies demonstrate considerable changes in the expression and functions of presynaptic proteins, attributed in part to direct effects of Aβ on the synaptic vesicle cycle (SVC) [55]. This effect of Aβ on the SVC is not surprising as the SVC is considered as both the prime site of Aβ production and toxicity [55]. However, insight into the role of TG2 in synaptic vesicle cycling and release is very limited. TGs are known to covalently modify synapsin, which binds to small synaptic vesicles and is involved in neurotransmitter release [56]. In addition, TG2 binds and crosslinks α-synuclein, a protein known for its role in synaptic vesicle budding, exacerbating alpha-synuclein’s toxicity [57]. Interestingly, in the present study we observed TG2 interactors involved in the synaptic vesicle pathway, e.g., RAB-3, dynamin, secretogranin, synaptotagmin and synaptosomol-associated proteins, suggesting that TG2 plays a more important role in the synaptic vesicle pathway than considered thus far. Apart from the cell adhesion- and synaptic vesicles-related pathways, an enlarged protein cluster related to mitochondrial energy metabolism was identified in networks of APP23 mice compared to WT, suggesting a link of TG2 with mitochondrial stress typical of Aβ-associated pathology. Indeed, mitochondrial dysfunction is a well-known phenomenon in AD, appearing as impaired energy metabolism, disrupted mitochondrial bioenergetics and genomic homeostasis, and abnormal fusion and fission (reviewed by Wang et al) [36]. In the context of the results of the current study it is therefore of interest to note that TG2 is also closely linked to mitochondria and mitochondrial functioning, as TG2 is localized in various brain cells at both the inner and outer mitochondrial membrane space and the matrix, and its crosslinking activity is associated with “mitochondrial disease” [58]. In fact, various mitochondrial proteins, e.g., G3PDH, Bax, ANT1, Prohibitin, Aconitase 2 and ATP Synthase Beta are interactors and substrates of TG2 specifically under pathological conditions [58].

Our comparative proteomics data are in line with the TG2 interactome data, as they also reveal alterations in proteins involved in vesicle trafficking and synaptic transmission release. Proteins that are part of these pathways, i.e., RAB1B (known to control intracellular membrane trafficking), and CPLX1 and 2 (which interact with SNAREs proteins in neurotransmitter release), were found to be upregulated in APP23 compared to APP23/TG2^−/−^ mice. Conversely, CYB1, involved in cGMP signaling-related long-term potentiation (LTP) underlying memory formation [59,60], and GNAZ consistently increased in the APP23/TG2^−/−^ brains, and decreased in APP23 brains compared to WT, suggesting that they are also associated with TG2-mediated APP23 pathology. Moreover, and in line with previous proteomic analysis of the APP23 mouse model, we also found Complexins and Guanine nucleotide-binding proteins significantly altered in the APP23 mouse model [37,38]. However, analysis of the TG2 interactors in the APP23 brain and concurrently of the APP23 proteome has also revealed that less than 1% of the TG2 partners emerged as increased in the AD-mimicking model in our all-round investigation, suggesting modification of a specific set of proteins in brain post-translationally by transglutaminase as part of the pathological process, rather than transcriptionally.

Altogether, by performing a comprehensive and unbiased analysis of the proteome and TG2 interactome of APP23 and WT animals, using TG2^−/−^ crossbred animals to exclude non-specific TG2 interactors, we found both known and novel TG2 interactors linked to Aβ pathology and related cellular processes, location and pathways in APP23 mice. Surprisingly, despite the elaborate biochemical and both human and murine post mortem studies linking TG2 to both the Aβ cascade and Aβ pathology, we here did not detect human or murine Aβ as a TG2 interactor. In addition, proteomics data revealed that Aβ (A4) levels changed independently from TG2 between APP23 and WT. This suggests that TG2 role in the Aβ cascade and/or pathology might be of a non-Aβ-related nature, and needs further exploring to unravel the mechanisms by which TG2 is involved in neuronal dysfunction and neurodegeneration in AD. Exploring the role of proteins involved in AD via a non-Aβ-centered approach might lead to a better understanding of AD pathophysiology, and open up new inroads to the development of novel and more effective strategies for treatment.

## Figures and Tables

**Table 1:** Proteins significantly interacting with TG2 in the extracellular space and plasma membrane, in the WT brain, APP23 brain or both phenotypes.

ID	TG2-Interactor Name	*p*-Value	Phenotype
TENR	Tenascin-R	1.5 × 10^−4^	WT
PTPRD	Receptor-type tyrosine-protein phosphatase delta	3.1 × 10^−3^	WT
RSU1	Ras suppressor protein 1	4.7 × 10^−3^	WT
CAD12	Cadherin-12	6.4 × 10^−3^	WT
NRCAM	Neuronal cell adhesion molecule	9.6 × 10^−3^	WT
S12A5	Solute carrier family 12 member 5	1.9 × 10^−2^	WT
SCN9A	Sodium channel protein type 9 subunit alpha	3.8 × 10^−2^	WT
SC6A1	Sodium- and chloride-dependent GABA transporter 1	2.9 × 10^−2^/8.0 × 10^−3^	WT/APP23
L1CAM	Neural cell adhesion molecule L1	3.2 × 10^−2^/2.4 × 10^−2^	WT/APP23
HEMO	Hemopexin	4.5 × 10^−2^/2.8 × 10^−4^	WT/APP23
PGCB	Brevican core protein	1.0 × 10^−5^	APP23
GRID2	Glutamate receptor ionotropic, delta-2	1.0 × 10^−5^	APP23
CA2D2	Voltage-dependent calcium channel subunit alpha-2/delta-2	1.8 × 10^−4^	APP23
SCG2	Secretogranin-2	1.6 × 10^−3^	APP23
AT2B1	Plasma membrane calcium-transporting ATPase 1	3.2 × 10^−3^	APP23
SEM3C	Semaphorin-3C	4.6 × 10^−3^	APP23
NRX2A	Neurexin-2	6.0 × 10^−3^	APP23
GPM6A	Neuronal membrane glycoprotein M6-a	1.5 × 10^−2^	APP23
MYPR	Myelin proteolipid protein	1.6 × 10^−2^	APP23
PLXB2	Plexin-B2	2.4 × 10^−2^	APP23
GRM3	Metabotropic glutamate receptor 3	5.5 × 10^−2^	APP23

The specificity of association with TG2 was evaluated by *z*-test analysis (*p* ≤ 0.05) of *n* = 5 animals per cohort, using the TG2^−/−^ mice as background control (as shown in Figure 3A,B). Proteins are denoted by UniProtKB protein entry name (ID) and full name, and they are listed according to the specificity of the interaction with TG2 (*p*-value). WT, TG2-associated proteins in WT brain; WT/APP23, TG2-associated proteins in both WT and APP23 brain; APP23, TG2-associated proteins in APP23 brain.

**Table 2:** Proteins significantly interacting with TG2 at the cell–matrix interface, in the WT brain, the APP23 brain or both phenotypes.

ID	TG2-Interactor Name	*p*-Value	Phenotype
HSP72	Heat shock-related 70 kDa protein 2	2.4 × 10^−15^	WT
ANK1	Ankyrin-1	4.7 × 10^−14^	WT
FIBG	Fibrinogen gamma chain	1.0 × 10^−11^	WT
TNIK	Traf2 and NCK-interacting protein kinase	1.5 × 10^−7^	WT
SRC8	Src substrate cortactin	9.4 × 10^−7^	WT
LRP1	Prolow-density lipoprotein receptor-related protein 1	2.3 × 10^−6^	WT
CYLD	Ubiquitin carboxyl-terminal hydrolase CYLD	2.9 × 10^−6^	WT
CPNE5	Copine-5	4.8 × 10^−6^	WT
ENPL	Endoplasmin	6.6 × 10^−6^	WT
CFAH	Complement factor H	2.5 × 10^−5^	WT
ASTN1	Astrotactin-1	2.6 × 10^−5^	WT
ACBG1	Long-chain-fatty-acid—CoA ligase ACSBG1	6.9 × 10^−5^	WT
FAS	Fatty acid synthase	4.2 × 10^−4^	WT
VATC1	V-type proton ATPase subunit C 1	5.3 × 10^−4^	WT
IDE	Insulin-degrading enzyme	6.8 × 10^−4^	WT
K2C1	Keratin, type II cytoskeletal 1	2.9 × 10^−3^	WT
SPB6	Serpin B6	3.5 × 10^−3^	WT
ADA10	Disintegrin and metalloproteinase domain-containing protein 10	4.2 × 10^−3^	WT
INP4A	Type I inositol 3,4-bisphosphate 4-phosphatase	6.9 × 10^−3^	WT
CYTC	Cystatin-C	7.7 × 10^−3^	WT
PICAL	Phosphatidylinositol-binding clathrin assembly protein	7.8 × 10^−3^	WT
HYEP	Epoxide hydrolase 1	8.8 × 10^−3^	WT
PDIA3	Protein disulfide-isomerase A3	1.0 × 10^−2^	WT
L2GL1	Lethal(2) giant larvae protein homolog 1	1.0 × 10^−2^	WT
CUL5	Cullin-5	1.1 × 10^−2^	WT
HEXB	Beta-hexosaminidase subunit beta	1.5 × 10^−2^	WT
CKAP5	Cytoskeleton-associated protein 5	1.5 × 10^−2^	WT
MSRA	Mitochondrial peptide methionine sulfoxide reductase	1.6 × 10^−2^	WT
APBB1	Amyloid-beta A4 precursor protein-binding family B member 1	2.0 × 10^−2^	WT
OGT1	UDP-N-acetylglucosamine—peptide N-acetylglucosaminyltransferase 110 kDa subunit	2.0 × 10^−2^	WT
ARBK1	Beta-adrenergic receptor kinase 1	2.4 × 10^−2^	WT
ABR	Active breakpoint cluster region-related protein	2.5 × 10^−2^	WT
RAB21	Ras-related protein Rab-21	2.6 × 10^−2^	WT
RHEB	GTP-binding protein Rheb	2.7 × 10^−2^	WT
ITPR1	Inositol 1,4,5-trisphosphate receptor type 1	2.7 × 10^−2^	WT
WASF1	Wiskott-Aldrich syndrome protein family member 1	2.8 × 10^−2^	WT
GARS	Glycine—tRNA ligase	3.5 × 10^−2^	WT
RACK1	Receptor of activated protein C kinase 1	3.5 × 10^−2^	WT
ACLY	ATP-citrate synthase	3.7 × 10^−2^	WT
SNAA	Alpha-soluble NSF attachment protein	3.8 × 10^−2^	WT
ATP5J	ATP synthase-coupling factor 6, mitochondrial	4.0 × 10^−2^	WT
E41L3	Band 4.1-like protein 3	4.0 × 10^−2^	WT
SOGA3	Protein SOGA3	4.4 × 10^−2^	WT
ALDR	Aldose reductase	4.6 × 10^−2^	WT
LYNX1	Ly-6/neurotoxin-like protein 1	4.8 × 10^−2^	WT
DAAM1	Disheveled-associated activator of morphogenesis 1	5.1 × 10^−12^/1.1 × 10^−4^	WT/APP23
PLAP	Phospholipase A-2-activating protein	3.2 × 10^−6^/2.6 × 10^−3^	WT/APP23
APOE	Apolipoprotein E	3.5 × 10^−5^/4.3 × 10^−8^	WT/APP23
RIN1	Ras and Rab interactor 1	1.3 × 10^−3^/2.9 × 10^−3^	WT/APP23
RIF1	Telomere-associated protein RIF1	9.7 × 10^−3^/2.8 × 10^−2^	WT/APP23
NDRG1	Protein NDRG1	1.9 × 10^−2^/1.0 × 10^−16^	WT/APP23
SYT2	Synaptotagmin-2	4.2 × 10^−2^/5.0 × 10^−2^	WT/APP23
ALBU	Serum albumin	5.5 × 10^−2^/2.8 × 10^−9^	WT/APP23
KCRB	Creatine kinase B-type	1.0 × 10^−16^	APP23
CRYAB	Alpha-crystallin B chain	1.0 × 10^−16^	APP23
1433F	14-3-3 protein eta	1.0 × 10^−16^	APP23
LANC2	LanC-like protein 2	2.4 × 10^−15^	APP23
GDIR1	Rho GDP-dissociation inhibitor 1	6.8 × 10^−12^	APP23
NRX1A	Neurexin-1	2.1 × 10^−11^	APP23
CAPS1	Calcium-dependent secretion activator 1	3.7 × 10^−11^	APP23
VATA	V-type proton ATPase catalytic subunit A	1.4 × 10^−9^	APP23
EF1A1	Elongation factor 1-alpha 1	3.5 × 10^−8^	APP23
MBP	Myelin basic protein	3.7 × 10^−8^	APP23
CADM2	Cell adhesion molecule 2	1.7 × 10^−6^	APP23
PCAT1	Lysophosphatidylcholine acyltransferase 1	2.3 × 10^−6^	APP23
NPTX1	Neuronal pentraxin-1	4.0 × 10^−6^	APP23
LYRIC	Protein LYRIC	4.0 × 10^−6^	APP23
PP4P1	Type 1 phosphatidylinositol 4,5-bisphosphate 4-phosphatase	5.7 × 10^−6^	APP23
CAH2	Carbonic anhydrase 2	7.4 × 10^−6^	APP23
ATPA	ATP synthase subunit alpha, mitochondrial	1.3 × 10^−5^	APP23
LSAMP	Limbic system-associated membrane protein	1.8 × 10^−5^	APP23
GLNA	Glutamine synthetase	2.4 × 10^−5^	APP23
SNP25	Synaptosomal-associated protein 25	3.6 × 10^−5^	APP23
PDIA6	Protein disulfide-isomerase A6	5.1 × 10^−5^	APP23
HS71A	Heat shock 70 kDa protein 1A	8.7 × 10^−5^	APP23
OCRL	Inositol polyphosphate 5-phosphatase OCRL-1	9.2 × 10^−5^	APP23
KPCB	Protein kinase C beta type	1.0 × 10^−4^	APP23
VINC	Vinculin	1.1 × 10^−4^	APP23
AT1B2	Sodium/potassium-transporting ATPase subunit beta-2	1.3 × 10^−4^	APP23
GNAO	Guanine nucleotide-binding protein G(o) subunit alpha	1.4 × 10^−4^	APP23
MYH10	Myosin-10	2.5 × 10^−4^	APP23
CDK5	Cyclin-dependent-like kinase 5	3.0 × 10^−4^	APP23
KCNC3	Potassium voltage-gated channel subfamily C member 3	3.1 × 10^−4^	APP23
NCKP1	Nck-associated protein 1	6.6 × 10^−4^	APP23
S10AD	Protein S100-A13	8.5 × 10^−4^	APP23
RAB3A	Ras-related protein Rab-3A	9.6 × 10^−4^	APP23
ADPRH	[Protein ADP-ribosylarginine] hydrolase	1.1 × 10^−3^	APP23
DYN1	Dynamin-1	1.4 × 10^−3^	APP23
SYT1	Synaptotagmin-1	1.7 × 10^−3^	APP23
PPP5	Serine/threonine-protein phosphatase 5	2.3 × 10^−3^	APP23
ENAH	Protein enabled homolog	2.4 × 10^−3^	APP23
RAP2B	Ras-related protein Rap-2b	3.2 × 10^−3^	APP23
KPCG	Protein kinase C gamma type	3.4 × 10^−3^	APP23
SH3G1	Endophilin-A2	3.5 × 10^−3^	APP23
MK03	Mitogen-activated protein kinase 3	4.2 × 10^−3^	APP23
PGK1	Phosphoglycerate kinase 1	4.6 × 10^−3^	APP23
GNAZ	Guanine nucleotide-binding protein G(z) subunit alpha	5.2 × 10^−3^	APP23
ANXA5	Annexin A5	6.8 × 10^−3^	APP23
GNB5	Guanine nucleotide-binding protein subunit beta-5	9.6 × 10^−3^	APP23
E41L1	Band 4.1-like protein 1	1.1 × 10^−2^	APP23
UCHL1	Ubiquitin carboxyl-terminal hydrolase isozyme L1	1.1 × 10^−2^	APP23
VATB2	V-type proton ATPase subunit B, brain isoform	1.2 × 10^−2^	APP23
GNAI2	Guanine nucleotide-binding protein G(i) subunit alpha-2	1.2 × 10^−2^	APP23
CAP2	Adenylyl cyclase-associated protein 2	1.2 × 10^−2^	APP23
DPYL2	Dihydropyrimidinase-related protein 2	1.5 × 10^−2^	APP23
RAB14	Ras-related protein Rab-14	1.5 × 10^−2^	APP23
PP2BA	Serine/threonine-protein phosphatase 2B catalytic subunit alpha isoform	1.7 × 10^−2^	APP23
HCN1	Potassium/sodium hyperpolarization-activated cyclic nucleotide-gated channel 1	1.8 × 10^−2^	APP23
PRAF3	PRA1 family protein 3	1.8 × 10^−2^	APP23
SPTN1	Spectrin alpha chain, non-erythrocytic 1	2.0 × 10^−2^	APP23
GRIA2	Glutamate receptor 2	3.3 × 10^−2^	APP23
SAHH2	S-adenosylhomocysteine hydrolase-like protein 1	3.8 × 10^−2^	APP23
COR1C	Coronin-1C	4.2 × 10^−2^	APP23
ENOG	Gamma-enolase	4.3 × 10^−2^	APP23
AT1A1	Sodium/potassium-transporting ATPase subunit alpha-1	4.7 × 10^−2^	APP23
NSF1C	NSFL1 cofactor p47	5.0 × 10^−2^	APP23

The specificity of association with TG2 was evaluated as explained in Table 1. WT, TG2-associated proteins in WT brain; WT/APP23, TG2-associated proteins in both WT and APP23 brain; APP23, TG2-associated proteins in APP23 brain.

**Table 3:** Proteins changed in APP23 compared to WT proteome. Positive log_2_FC indicates upregulated proteins; negative log_2_FC indicates downregulated proteins.

ID	Name	log_2_FC (APP23/WT)	Confidence
ELAV4	ELAV-like protein 4	3.93	0.55
TBCEL	Tubulin-specific chaperone cofactor E-like protein	2.84	0.56
ATP5I	ATP synthase subunit e, mitochondrial	2.56	0.60
GCYB1	Guanylate cyclase soluble subunit beta-1	2.47	0.71
SNX4	Sorting nexin-4	2.34	0.58
PSMD3	26S proteasome non-ATPase regulatory subunit 3	1.31	0.68
A4	Amyloid beta A4 protein	1.18	0.78
CPLX2	Complexin-2	1.09	0.58
CPLX1	Complexin-1	1.08	0.63
RAB1B	Ras-related protein Rab-1B	1.03	0.70
BAG3	BAG family molecular chaperone regulator 3	0.91	0.55
CAH8	Carbonic anhydrase-related protein	0.76	0.67
HBB1	Hemoglobin subunit beta-1	0.70	0.56
ABHGA	Protein ABHD16A	0.60	0.65
CAZA2	F-actin-capping protein subunit alpha-2	0.57	0.65
PRVA	Parvalbumin alpha	0.47	0.68
NFH	Neurofilament heavy polypeptide	0.47	0.67
HNRPU	Heterogeneous nuclear ribonucleoprotein U	0.41	0.55
OCAD2	OCIA domain-containing protein 2	0.38	0.63
L1CAM	Neural cell adhesion molecule L1	0.37	0.60
FA49B	Protein FAM49B	0.33	0.66
IPP2	Protein phosphatase inhibitor 2	0.33	0.63
GNAZ	Guanine nucleotide-binding protein G(z) subunit alpha	0.32	0.56
S4A4	Electrogenic sodium bicarbonate cotransporter 1	0.28	0.56
NEUG	Neurogranin	0.28	0.55
CANB1	Calcineurin subunit B type 1	0.27	0.55
NAC2	Sodium/calcium exchanger 2	−0.48	0.81
MP2K1	Dual specificity mitogen-activated protein kinase kinase 1	−0.49	0.57
NPTXR	Neuronal pentraxin receptor	−0.53	0.55
PC4L1	Purkinje cell protein 4-like protein 1	−0.54	0.82

Proteins changed at confidence ≥ 55% are listed according to log_2_(FC) level. APP23/WT ratio expressed as log_2_FC (log_2_(APP23/WT)) is here shown.

**Table 4:** Proteins changed in APP23 compared to APP23/TG2^−/−^ proteome. Positive log_2_FC indicates upregulated proteins; negative log_2_FC indicates downregulated proteins.

ID	Name	log_2_FC (APP23/APP23 TG2^−/−^)	Confidence
UCHL1	Ubiquitin carboxyl-terminal hydrolase isozyme L1	6.02	0.58
MATR3	Matrin-3	3.32	0.57
MDHC	Malate dehydrogenase, cytoplasmic	1.78	0.55
MAAI	Maleylacetoacetate isomerase	1.59	0.70
CPLX2	Complexin-2	1.41	0.57
S4A4	Electrogenic sodium bicarbonate cotransporter 1	1.21	0.57
CPLX1	Complexin-1	1.16	0.59
RAB1B	Ras-related protein Rab-1B	1.01	0.71
HBB1	Hemoglobin subunit beta-1	0.77	0.66
NDKB	Nucleoside diphosphate kinase B	0.43	0.72
PPME1	Protein phosphatase methylesterase 1	0.43	0.62
GNAZ	Guanine nucleotide-binding protein G(z) subunit alpha	−0.37	0.55
OGT1	UDP-N-acetylglucosamine—peptide N-acetylglucosaminyltransferase 110 kDa subunit	−0.45	0.55
CLPP	ATP-dependent Clp protease proteolytic subunit, mitochondrial	−0.55	0.63
F210A	Protein FAM210A	−0.56	0.67
RL29	60S ribosomal protein L29	−0.74	0.56
KPCG	Protein kinase C gamma type	−0.94	0.72
PGM2L	Glucose 1,6-bisphosphate synthase	−0.94	0.72
UBE2N	Ubiquitin-conjugating enzyme E2 N	−1.00	0.79
RS9	40S ribosomal protein S9	−1.25	0.77
MECR	Trans-2-enoyl-CoA reductase, mitochondrial	−1.48	0.56
VAT1L	Synaptic vesicle membrane protein VAT-1 homolog-like	−1.49	0.56
RAB3B	Ras-related protein Rab-3B	−1.75	0.55
GUAA	GMP synthase [glutamine-hydrolyzing]	−2.23	0.62
GCYB1	Guanylate cyclase soluble subunit beta-1	−2.39	0.56
GMFB	Glia maturation factor beta	−2.57	0.58
THIKA	3-ketoacyl-CoA thiolase A, peroxisomal	−2.97	0.64
2ABG	Serine/threonine-protein phosphatase 2A 55 kDa regulatory subunit B gamma isoform	−3.43	0.61
MYDGF	Myeloid-derived growth factor	−3.59	0.57

The APP23 and APP23/TG2^−/−^ proteomes were resolved by SWATH acquisition MS as described in the Methods. Proteins changed at confidence ≥ 55% are listed according to log_2_(FC) level. APP23/APP23 TG2^−/−^ ratio was calculated by SCIEX OneOmics cloud processing software. The protein peak area variation expressed as log_2_FC (log_2_(APP23/APP23 TG2^−/−^)) is here shown.

**Table 5:** Proteins changed in APP23/TG2^−/−^ compared to WT/TG2^−/−^ proteome. Positive log_2_FC indicates upregulated proteins; negative log_2_FC indicates downregulated proteins.

ID	Name	log_2_FC (APP23 TG2^−/−^/WT TG2^−/−^)	Confidence
GNAI3	Guanine nucleotide-binding protein G(k) subunit alpha	3.55	0.56
MACF1	Microtubule-actin crosslinking factor 1	3.46	0.57
FN3K	Fructosamine-3-kinase	3.18	0.78
THIKA	3-ketoacyl-CoA thiolase A, peroxisomal	3.08	0.62
SYMC	Methionine—tRNA ligase, cytoplasmic	3.06	0.62
CPSF6	Cleavage and polyadenylation specificity factor subunit 6	2.75	0.62
NFU1	NFU1 iron-sulfur cluster scaffold homolog, mitochondrial	2.45	0.58
ESYT1	Extended synaptotagmin-1	2.40	0.71
ENOG	Gamma-enolase	2.07	0.60
SEP6	Septin-6	1.62	0.60
CLPP	ATP-dependent Clp protease proteolytic subunit, mitochondrial	1.49	0.88
A4	Amyloid beta A4 protein	1.26	0.62
NCLN	Nicalin	0.92	0.55
UCRI	Cytochrome b-c1 complex subunit Rieske, mitochondrial	0.83	0.59
NFM	Neurofilament medium polypeptide	0.73	0.79
UBE2N	Ubiquitin-conjugating enzyme E2 N	0.68	0.65
KPCG	Protein kinase C gamma type	0.58	0.67
CAMP2	Calmodulin-regulated spectrin-associated protein 2	0.50	0.56
APOD	Apolipoprotein D	0.49	0.56
BRSK1	Serine/threonine-protein kinase BRSK1	0.46	0.67
SCPDL	Saccharopine dehydrogenase-like oxidoreductase	0.39	0.68
TNPO1	Transportin-1	0.36	0.56
GLU2B	Glucosidase 2 subunit beta	0.16	0.60
STMN1	Stathmin	−0.25	0.63
GPM6A	Neuronal membrane glycoprotein M6-a	−0.30	0.57
MPP2	MAGUK p55 subfamily member 2	−0.36	0.63
NEUG	Neurogranin	−0.49	0.68
OTU6B	OTU domain-containing protein 6B	−0.49	0.69
PC4L1	Purkinje cell protein 4-like protein 1	−0.51	0.83
CH10	10 kDa heat shock protein, mitochondrial	−0.56	0.64
HBB1	Hemoglobin subunit beta-1	−0.60	0.71
MDHC	Malate dehydrogenase, cytoplasmic	−0.82	0.57
SNG1	Synaptogyrin-1	−0.92	0.55
KCAB2	Voltage-gated potassium channel subunit beta-2	−0.94	0.66
MAAI	Maleylacetoacetate isomerase	−1.37	0.65
TDRKH	Tudor and KH domain-containing protein	−1.66	0.57
ENOPH	Enolase-phosphatase E1	−2.11	0.60
SARM1	Sterile alpha and TIR motif-containing protein 1	−2.21	0.62
PZP	Pregnancy zone protein	−2.38	0.62
PUR6	Multifunctional protein ADE2	−4.39	0.70

Proteins changed at confidence ≥55% are listed according to log_2_(FC) level. APP23 TG2^−/−^/WT TG2^−/−^ ratio expressed as log_2_FC (log_2_(APP23 TG2^−/−^/WT TG2^−/−^)) is here shown.

**Table 6:** Proteins changed in WT compared to WT/TG2^−/−^ proteome. Positive log_2_FC indicates upregulated proteins; negative log_2_FC indicates downregulated proteins.

ID	Name	log_2_FC (WT/WT TG2^−/−^)	Confidence
CPSF6	Cleavage and polyadenylation specificity factor subunit 6	3.76	0.68
GNAI3	Guanine nucleotide-binding protein G(k) subunit alpha	3.75	0.55
PADI2	Protein-arginine deiminase type-2	3.59	0.59
TOLIP	Toll-interacting protein	3.53	0.61
SYMC	Methionine—tRNA ligase, cytoplasmic	3.46	0.60
ECHD1	Ethylmalonyl-CoA decarboxylase	2.54	0.60
HSDL1	Inactive hydroxysteroid dehydrogenase-like protein 1	2.50	0.55
AGRB1	Brain-specific angiogenesis inhibitor 1	2.49	0.55
NFU1	NFU1 iron-sulfur cluster scaffold homolog, mitochondrial	2.21	0.66
FBX41	F-box only protein 41	1.17	0.64
APOD	Apolipoprotein D	0.68	0.57
SNAA	Alpha-soluble NSF attachment protein	0.47	0.58
HEBP1	Heme-binding protein 1	0.27	0.56
RRAGC	Ras-related GTP-binding protein C	0.27	0.55
DTD1	D-tyrosyl-tRNA(Tyr) deacylase 1	0.23	0.68
RL6	60S ribosomal protein L6	0.21	0.56
CORO7	Coronin-7	−0.29	0.63
AN32A	Acidic leucine-rich nuclear phosphoprotein 32 family member A	−0.35	0.64
HNRPU	Heterogeneous nuclear ribonucleoprotein U	−0.43	0.73
CAH8	Carbonic anhydrase-related protein	−0.47	0.55
MBP	Myelin basic protein	−0.61	0.75
HBB1	Hemoglobin subunit beta-1	−0.62	0.79
MYEF2	Myelin expression factor 2	−0.63	0.57
S20A2	Sodium-dependent phosphate transporter 2	−0.79	0.80
KCAB2	Voltage-gated potassium channel subunit beta-2	−0.85	0.61
RL35A	60S ribosomal protein L35a	−0.94	0.55
RS28	40S ribosomal protein S28	−1.01	0.67
LSM6	U6 snRNA-associated Sm-like protein LSm6	−1.02	0.57
RL29	60S ribosomal protein L29	−1.03	0.78
AN32E	Acidic leucine-rich nuclear phosphoprotein 32 family member E	−1.03	0.61
RS9	40S ribosomal protein S9	−1.16	0.61
SNG1	Synaptogyrin-1	−1.23	0.57
SARM1	Sterile alpha and TIR motif-containing protein 1	−2.27	0.65
EXOG	Nuclease EXOG, mitochondrial	−3.22	0.56
RHG23	Rho GTPase-activating protein 23	−3.38	0.63
ROCK2	Rho-associated protein kinase 2	−3.61	0.61

Proteins changed at confidence ≥ 55% are listed according to log_2_(FC) level. WT/WT TG2^−/−^ ratio expressed as log_2_FC (log_2_(WT/WT TG2^−/−^)) is here shown.

## Data Availability

The mass spectrometry proteomics data were deposited to the ProteomeXchange Consortium via the PRIDE partner repository with the dataset identifier “PXD030354”.

## Supplementary Materials

The following are available online at https://www.mdpi.com/article/10.3390/cells11030389/s1, Supplementary Table S1: Details of antibodies used for IHC and IF; Supplementary Table S2: Details of primers used for semi-quantitative RT-PCR; Supplementary Table S3: Full list of proteins significantly interacting with TG2 in the WT brain, APP23 brain or both phenotypes.
