# Supplementary material for: The Transglutaminase-2 Interactome in the APP23 Mouse Model of Alzheimer’s Disease
Source: Cells. 2022 Jan 24;11(3):389. doi: 10.3390/cells11030389 (PMC8834516; doi:10.3390/cells11030389)
Supplement: Supplementary file 1 [file cells-11-00389-s001.zip › cells-1532521-supplementary.pdf]

Supplementary files

Supplementary Table S1: Details of antibodies used for IHC and IF.

Antigen	Primary antibody	Dilution	Raised in	Fixation	Company
Amyloid- Beta	Human Aβ (715800)	1/400	Rabbit	Acetone	Invitrogen. Carlsbad, CA, USA
Astrocytes	Bovine glial fibrillary acidic protein antibody	1/400	Rabbit	Acetone	DAKO. Santa Clara, CA, USA
Microglia	CD45- Mouse protein tyrosine phosphatase receptor type C	1/100	Rat	Acetone	Gift cell biology VUMC
Hyper-phosphorylated tau	tau protein phosphorylated at both serine 202 and threonine 205	1/2000	Mouse	PFA	Thermo Fischer scientific, Waltham, MA, USA
TG2	Guinea pig TG2 (06471)	1/4000	Goat	Acetone	Millipore, Temecula, CA, USA

Supplementary Table S2: Details of primers used for semi-quantitative RT-PCR.

Gene	Forward	Reverse
**mouse HPRT1**	CTCATGGACTGATTATGGACAGGAC	GCAGGTCAGCAAAGAACTTATAGCC
**human APP**	ACACAGAAAACGAAGTTGAGCC	AATTCTGCATCCAGATTCACTTCAGA
**mouse TGM1**	ACCAGCAGTGGCATCTTC	ATGAAAGGTGTGTCATACTTC
**mouse TGM2**	GCCATGGTCAACTGCAATG	AGAATGTCCACACTGCCAATC
**mouse TGM3**	ACATCAGCACCAAGGCAGTAGG	CCTCGAAGATGTTGCGCCGAAA
**mouse F13A1**	CAGCAATGGTGAATGCCAAGGA	GCTGATGGAGGGATGCCGTA

Oligonucleotide PCR primers are listed in the 5’→ 3’ direction

Supplementary Table S3: Full list of proteins significantly interacting with TG2 in the WT brain, APP23 brain or both phenotypes.

ID	TG2-interactor name	p-value	Phenotype
1433F	14-3-3 protein eta	0.00E+00	APP23
AATC	Aspartate aminotransferase, cytoplasmic	0.00E+00	APP23
AL1B1	Aldehyde dehydrogenase X, mitochondrial	0.00E+00	APP23
ARGL1	Arginine and glutamate-rich protein 1	0.00E+00	APP23
AT5F1	ATP synthase F(0) complex subunit B1, mitochondrial	0.00E+00	APP23
CRYAB	Alpha-crystallin B chain	0.00E+00	APP23
GRID2	Glutamate receptor ionotropic, delta-2	0.00E+00	APP23
KCRB	Creatine kinase B-type	0.00E+00	APP23
KV5AD	Ig kappa chain V-V region HP 123E6	0.00E+00	APP23
PGCB	Brevican core protein	0.00E+00	APP23
RS17	40S ribosomal protein S17	0.00E+00	APP23
SYNE1	Nesprin-1	0.00E+00	APP23
TPM3	Tropomyosin alpha-3 chain	0.00E+00	APP23
MYO5A	Unconventional myosin-Va	0.00E+00	WT
NDUS3	NADH dehydrogenase [ubiquinone] iron-sulfur protein 3, mitochondrial	0.00E+00	WT
RS16	40S ribosomal protein S16	0.00E+00	WT
TECR	Very-long-chain enoyl-CoA reductase	0.00E+00	WT
LANC2	LanC-like protein 2	2.44E-15	APP23
HSP72	Heat shock-related 70 kDa protein 2	2.44E-15	WT
TBB3	Tubulin beta-3 chain	2.44E-15	WT
PSB1	Proteasome subunit beta type-1	3.49E-14	WT/APP23
ANK1	Ankyrin-1	4.72E-14	WT
CYC	Cytochrome c, somatic	9.15E-14	APP23
MED23	Mediator of RNA polymerase II transcription subunit 23	4.80E-13	APP23
RL29	60S ribosomal protein L29	3.04E-12	APP23
DAAM1	Disheveled-associated activator of morphogenesis 1	5.07E-12	WT/APP23
GDIR1	Rho GDP-dissociation inhibitor 1	6.76E-12	APP23
FIBG	Fibrinogen gamma chain	1.02E-11	WT
ATPG	ATP synthase subunit gamma, mitochondrial	1.09E-11	APP23
PPCEL	Prolyl endopeptidase-like	1.79E-11	APP23
NRX1A	Neurexin-1	2.09E-11	APP23
CLH1	Clathrin heavy chain 1	3.21E-11	APP23
CAPS1	Calcium-dependent secretion activator 1	3.69E-11	APP23
KKCC2	Calcium/calmodulin-dependent protein kinase kinase 2	6.98E-11	APP23
1433T	14-3-3 protein theta	7.89E-10	APP23
VATA	V-type proton ATPase catalytic subunit A	1.35E-09	APP23
RL24	60S ribosomal protein L24	7.26E-09	APP23
CTL1	Choline transporter-like protein 1	9.93E-09	APP23
PURA	Transcriptional activator protein Pur-alpha	2.23E-08	APP23
EF1A1	Elongation factor 1-alpha 1	3.51E-08	APP23
MBP	Myelin basic protein	3.69E-08	APP23
SEPT7	Septin-7	4.47E-08	APP23

PURA1	Adenylosuccinate synthetase isozyme 1	5.65E-08	WT
TNIK	Traf2 and NCK-interacting protein kinase	1.45E-07	WT
ITPA	Inosine triphosphate pyrophosphatase	1.47E-07	WT
HIBCH	3-hydroxyisobutyryl-CoA hydrolase, mitochondrial	2.23E-07	APP23
CDS2	Phosphatidate cytidylyltransferase 2	5.10E-07	APP23
F210A	Protein FAM210A	6.83E-07	WT
SRC8	Src substrate cortactin	9.39E-07	WT
HMCS1	Hydroxymethylglutaryl-CoA synthase, cytoplasmic	9.48E-07	WT
SH3L3	SH3 domain-binding glutamic acid-rich-like protein 3	1.01E-06	APP23
PDK3	[Pyruvate dehydrogenase (acetyl-transferring)] kinase isozyme 3, mitochondrial	1.18E-06	APP23
AL4A1	Delta-1-pyrroline-5-carboxylate dehydrogenase, mitochondrial	1.38E-06	APP23
GLSK	Glutaminase kidney isoform, mitochondrial	1.57E-06	WT
CADM2	Cell adhesion molecule 2	1.68E-06	APP23
LRP1	Prolow-density lipoprotein receptor-related protein 1	2.31E-06	WT
PCAT1	Lysophosphatidylcholine acyltransferase 1	2.33E-06	APP23
KV2A6	Ig kappa chain V-II region 7S34.1	2.46E-06	APP23
CYLD	Ubiquitin carboxyl-terminal hydrolase CYLD	2.88E-06	WT
IPO9	Importin-9	2.92E-06	APP23
PLAP	Phospholipase A-2-activating protein	3.24E-06	WT/APP23
NPTX1	Neuronal pentraxin-1	3.95E-06	APP23
GLYM	Serine hydroxymethyltransferase, mitochondrial	3.97E-06	APP23
LYRIC	Protein LYRIC	4.00E-06	APP23
BDH	D-beta-hydroxybutyrate dehydrogenase, mitochondrial	4.37E-06	WT
CPNE5	Copine-5	4.79E-06	WT
CSKI1	Caskin-1	5.05E-06	APP23
DMXL2	DmX-like protein 2	5.71E-06	WT/APP23
PP4P1	Type 1 phosphatidylinositol 4,5-bisphosphate 4-phosphatase	5.71E-06	APP23
ENPL	Endoplasmin	6.63E-06	WT
CAH2	Carbonic anhydrase 2	7.36E-06	APP23
GHC1	Mitochondrial glutamate carrier 1	8.00E-06	APP23
KPRA	Phosphoribosyl pyrophosphate synthase-associated protein 1	1.18E-05	WT
EFTU	Elongation factor Tu, mitochondrial	1.19E-05	APP23
ATPA	ATP synthase subunit alpha, mitochondrial	1.32E-05	APP23
KCY	UMP-CMP kinase	1.53E-05	WT
LSAMP	Limbic system-associated membrane protein	1.82E-05	APP23
UGPA	UTP--glucose-1-phosphate uridylyltransferase	2.11E-05	APP23
GLNA	Glutamine synthetase	2.39E-05	APP23
CFAH	Complement factor H	2.50E-05	WT
ASTN1	Astrotactin-1	2.63E-05	WT
AP1B1	AP-1 complex subunit beta-1	2.80E-05	APP23
APOE	Apolipoprotein E	3.48E-05	WT/APP23
SNP25	Synaptosomal-associated protein 25	3.60E-05	APP23
OGFR	Opioid growth factor receptor	4.29E-05	APP23
SCMC2	Calcium-binding mitochondrial carrier protein SCaMC-2	4.31E-05	WT/APP23
IGKC	Immunoglobulin kappa constant	4.49E-05	APP23
PDIA6	Protein disulfide-isomerase A6	5.13E-05	APP23

GSTM5	Glutathione S-transferase Mu 5	5.70E-05	APP23
TCAL5	Transcription elongation factor A protein-like 5	5.81E-05	APP23
MA7D1	MAP7 domain-containing protein 1	6.01E-05	APP23
ACBG1	Long-chain-fatty-acid--CoA ligase ACSBG1	6.88E-05	WT
MAOX	NADP-dependent malic enzyme	7.79E-05	APP23
DCTN1	Dynactin subunit 1	8.24E-05	WT
HS71A	Heat shock 70 kDa protein 1A	8.68E-05	APP23
OCRL	Inositol polyphosphate 5-phosphatase OCRL-1	9.20E-05	APP23
RUVB2	RuvB-like 2	9.81E-05	WT/APP23
KPCB	Protein kinase C beta type	1.04E-04	APP23
VINC	Vinculin	1.07E-04	APP23
HVM10	Ig heavy chain V region 1-62-3	1.08E-04	APP23
H10	Histone H1.0	1.09E-04	WT
H14	Histone H1.4	1.24E-04	APP23
AT1B2	Sodium/potassium-transporting ATPase subunit beta-2	1.26E-04	APP23
GNAO	Guanine nucleotide-binding protein G(o) subunit alpha	1.43E-04	APP23
TENR	Tenascin-R	1.46E-04	WT
PGM2L	Glucose 1,6-bisphosphate synthase	1.46E-04	WT
THOP1	Thimet oligopeptidase	1.46E-04	APP23
CA2D2	Voltage-dependent calcium channel subunit alpha-2/delta-2	1.84E-04	APP23
IP3KA	Inositol-trisphosphate 3-kinase A	2.27E-04	WT
DEST	Destrin	2.34E-04	APP23
SFR1	Swi5-dependent recombination DNA repair protein 1 homolog	2.47E-04	WT
MYH10	Myosin-10	2.47E-04	APP23
DLDH	Dihydrolipoyl dehydrogenase, mitochondrial	2.53E-04	APP23
RL13	60S ribosomal protein L13	2.57E-04	WT/APP23
KV4A1	Ig kappa chain V-IV region S107B	2.60E-04	APP23
CDK5	Cyclin-dependent-like kinase 5	3.01E-04	APP23
CH10	10 kDa heat shock protein, mitochondrial	3.10E-04	APP23
KCNC3	Potassium voltage-gated channel subfamily C member 3	3.13E-04	APP23
AL7A1	Alpha-aminoadipic semialdehyde dehydrogenase	3.31E-04	APP23
EPMIP	EPM2A-interacting protein 1	3.40E-04	WT
FAS	Fatty acid synthase	4.21E-04	WT
KV5A1	Ig kappa chain V19-17	4.72E-04	APP23
PTN23	Tyrosine-protein phosphatase non-receptor type 23	4.77E-04	APP23
SAHH	Adenosylhomocysteinase	4.91E-04	APP23
GOGA7	Golgin subfamily A member 7	5.01E-04	APP23
VATC1	V-type proton ATPase subunit C 1	5.25E-04	WT
NIPS1	Protein NipSnap homolog 1	5.28E-04	WT/APP23
SBP1	Methanethiol oxidase	5.92E-04	WT
PHF24	PHD finger protein 24	6.03E-04	APP23
NDUA4	Cytochrome c oxidase subunit NDUFA4	6.06E-04	APP23
KV2A7	Ig kappa chain V-II region 26-10	6.12E-04	APP23
DECR	2,4-dienoyl-CoA reductase, mitochondrial	6.40E-04	WT
TCPA	T-complex protein 1 subunit alpha	6.58E-04	WT
NCKP1	Nck-associated protein 1	6.59E-04	APP23

IDE	Insulin-degrading enzyme	6.76E-04	WT
NFL	Neurofilament light polypeptide	7.14E-04	APP23
COX41	Cytochrome c oxidase subunit 4 isoform 1, mitochondrial	7.47E-04	APP23
TBB4A	Tubulin beta-4A chain	8.23E-04	APP23
S10AD	Protein S100-A13	8.46E-04	APP23
USP9X	Probable ubiquitin carboxyl-terminal hydrolase FAF-X	8.93E-04	WT
RAB3A	Ras-related protein Rab-3A	9.62E-04	APP23
USMG5	Up-regulated during skeletal muscle growth protein 5	9.67E-04	WT
ADPRH	[Protein ADP-ribosylarginine] hydrolase	1.05E-03	APP23
LRC59	Leucine-rich repeat-containing protein 59	1.18E-03	APP23
RIN1	Ras and Rab interactor 1	1.26E-03	WT/APP23
CAMP3	Calmodulin-regulated spectrin-associated protein 3	1.28E-03	APP23
DYN1	Dynamin-1	1.43E-03	APP23
FA98B	Protein FAM98B	1.53E-03	APP23
CNDP2	Cytosolic non-specific dipeptidase	1.59E-03	WT
SCG2	Secretogranin-2	1.65E-03	APP23
SYT1	Synaptotagmin-1	1.65E-03	APP23
HEMH	Ferrochelatase, mitochondrial	1.79E-03	WT
CMTD1	Catechol O-methyltransferase domain-containing protein 1	1.85E-03	APP23
K1C42	Keratin, type I cytoskeletal 42	1.86E-03	APP23
NDUS2	NADH dehydrogenase [ubiquinone] iron-sulfur protein 2, mitochondrial	1.96E-03	WT
RAB7A	Ras-related protein Rab-7a	1.99E-03	APP23
RL7	60S ribosomal protein L7	2.02E-03	WT
TRI32	E3 ubiquitin-protein ligase TRIM32	2.03E-03	WT
TIM44	Mitochondrial import inner membrane translocase subunit TIM44	2.19E-03	WT
SMD3	Small nuclear ribonucleoprotein Sm D3	2.19E-03	APP23
1433G	14-3-3 protein gamma	2.26E-03	WT
PPP5	Serine/threonine-protein phosphatase 5	2.30E-03	APP23
MTMR5	Myotubularin-related protein 5	2.33E-03	APP23
ATPD	ATP synthase subunit delta, mitochondrial	2.37E-03	APP23
ENAH	Protein enabled homolog	2.42E-03	APP23
QORL2	Quinone oxidoreductase-like protein 2	2.54E-03	WT/APP23
GGCT	Gamma-glutamylcyclotransferase	2.62E-03	APP23
XPO7	Exportin-7	2.64E-03	APP23
NDUAA	NADH dehydrogenase [ubiquinone] 1 alpha subcomplex subunit 10, mitochondrial	2.68E-03	APP23
DYHC1	Cytoplasmic dynein 1 heavy chain 1	2.83E-03	APP23
THIM	3-ketoacyl-CoA thiolase, mitochondrial	2.86E-03	APP23
K22E	Keratin, type II cytoskeletal 2 epidermal	2.89E-03	APP23
K2C1	Keratin, type II cytoskeletal 1	2.91E-03	WT
PTPRD	Receptor-type tyrosine-protein phosphatase delta	3.12E-03	WT
RAP2B	Ras-related protein Rap-2b	3.15E-03	APP23
CBR4	Carbonyl reductase family member 4	3.20E-03	WT
AT2B1	Plasma membrane calcium-transporting ATPase 1	3.24E-03	APP23
CY1	Cytochrome c1, heme protein, mitochondrial	3.31E-03	APP23
KPCG	Protein kinase C gamma type	3.35E-03	APP23
HVM32	Ig heavy chain V-III region J606	3.40E-03	WT

SH3G1	Endophilin-A2	3.45E-03	APP23
SPB6	Serpin B6	3.46E-03	WT
PSD12	26S proteasome non-ATPase regulatory subunit 12	3.85E-03	WT
ADA10	Disintegrin and metalloproteinase domain-containing protein 10	4.18E-03	WT
INF2	Inverted formin-2	4.23E-03	WT
MK03	Mitogen-activated protein kinase 3	4.23E-03	APP23
TXTP	Tricarboxylate transport protein, mitochondrial	4.46E-03	APP23
PGK1	Phosphoglycerate kinase 1	4.57E-03	APP23
SEM3C	Semaphorin-3C	4.58E-03	APP23
TOM34	Mitochondrial import receptor subunit TOM34	4.61E-03	WT/APP23
SYRC	Arginine--tRNA ligase, cytoplasmic	4.64E-03	WT/APP23
TRIO	Triple functional domain protein	4.70E-03	APP23
NLTP	Non-specific lipid-transfer protein	4.74E-03	APP23
RSU1	Ras suppressor protein 1	4.75E-03	WT
K1C10	Keratin, type I cytoskeletal 10	4.76E-03	APP23
CX7A2	Cytochrome c oxidase subunit 7A2, mitochondrial	4.93E-03	WT/APP23
CP250	Centrosome-associated protein CEP250	5.15E-03	WT
GNAZ	Guanine nucleotide-binding protein G(z) subunit alpha	5.24E-03	APP23
GABT	4-aminobutyrate aminotransferase, mitochondrial	5.76E-03	WT
ABHGA	Protein ABHD16A	5.93E-03	APP23
CLVS1	Clavesin-1	5.95E-03	WT/APP23
NRX2A	Neurexin-2	6.00E-03	APP23
PYC	Pyruvate carboxylase, mitochondrial	6.15E-03	WT
DNJB1	DnaJ homolog subfamily B member 1	6.16E-03	WT
KV3A4	Ig kappa chain V-III region 50S10.1	6.30E-03	APP23
CAD12	Cadherin-12	6.42E-03	WT
RS13	40S ribosomal protein S13	6.68E-03	WT
ANXA5	Annexin A5	6.80E-03	APP23
INP4A	Type I inositol 3,4-bisphosphate 4-phosphatase	6.88E-03	WT
CUL2	Cullin-2	6.91E-03	APP23
SCAM1	Secretory carrier-associated membrane protein 1	7.08E-03	APP23
EIF3D	Eukaryotic translation initiation factor 3 subunit D	7.59E-03	WT
CYTC	Cystatin-C	7.67E-03	WT
VPS35	Vacuolar protein sorting-associated protein 35	7.76E-03	APP23
DHX15	Pre-mRNA-splicing factor ATP-dependent RNA helicase DHX15	7.78E-03	APP23
PICAL	Phosphatidylinositol-binding clathrin assembly protein	7.81E-03	WT
SYSC	Serine--tRNA ligase, cytoplasmic	7.84E-03	APP23
VPS18	Vacuolar protein sorting-associated protein 18 homolog	7.90E-03	WT
ATLA1	Atlastin-1	8.09E-03	WT
RAN	GTP-binding nuclear protein Ran	8.14E-03	APP23
PSMD8	26S proteasome non-ATPase regulatory subunit 8	8.58E-03	WT
GSTA4	Glutathione S-transferase A4	8.78E-03	WT/APP23
HYEP	Epoxide hydrolase 1	8.81E-03	WT
TPC11	Trafficking protein particle complex subunit 11	8.81E-03	APP23
PRUN1	Exopolyphosphatase PRUNE1	9.35E-03	APP23
RPN1	Dolichyl-diphosphooligosaccharide--protein glycosyltransferase subunit 1	9.50E-03	APP23

CYFP2	Cytoplasmic FMR1-interacting protein 2	9.55E-03	WT
GNB5	Guanine nucleotide-binding protein subunit beta-5	9.56E-03	APP23
NRCAM	Neuronal cell adhesion molecule	9.64E-03	WT
RIF1	Telomere-associated protein RIF1	9.66E-03	WT/APP23
SCOT1	Succinyl-CoA:3-ketoacid coenzyme A transferase 1, mitochondrial	9.68E-03	WT
H13	Histone H1.3	9.84E-03	APP23
SYHC	Histidine--tRNA ligase, cytoplasmic	1.00E-02	APP23
PDIA3	Protein disulfide-isomerase A3	1.01E-02	WT
L2GL1	Lethal(2) giant larvae protein homolog 1	1.01E-02	WT
PCCA	Propionyl-CoA carboxylase alpha chain, mitochondrial	1.05E-02	WT
CUL5	Cullin-5	1.07E-02	WT
SUCB1	Succinate--CoA ligase [ADP-forming] subunit beta, mitochondrial	1.07E-02	APP23
CE170	Centrosomal protein of 170 kDa	1.08E-02	APP23
CA2D1	Voltage-dependent calcium channel subunit alpha-2/delta-1	1.08E-02	APP23
CALX	Calnexin	1.11E-02	WT
E41L1	Band 4.1-like protein 1	1.12E-02	APP23
ML12B	Myosin regulatory light chain 12B	1.12E-02	WT/APP23
GDIB	Rab GDP dissociation inhibitor beta	1.13E-02	APP23
UCHL1	Ubiquitin carboxyl-terminal hydrolase isozyme L1	1.14E-02	APP23
KV2A5	Ig kappa chain V-II region 17S29.1	1.18E-02	APP23
K22O	Keratin, type II cytoskeletal 2 oral	1.18E-02	APP23
VATB2	V-type proton ATPase subunit B, brain isoform	1.19E-02	APP23
F162A	Protein FAM162A	1.22E-02	WT
GNAI2	Guanine nucleotide-binding protein G(i) subunit alpha-2	1.24E-02	APP23
CAP2	Adenylyl cyclase-associated protein 2	1.24E-02	APP23
IGSF8	Immunoglobulin superfamily member 8	1.28E-02	APP23
TXD12	Thioredoxin domain-containing protein 12	1.30E-02	WT/APP23
ABD12	Monoacylglycerol lipase ABHD12	1.32E-02	WT
NQO2	Ribosyldihydronicotinamide dehydrogenase [quinone]	1.32E-02	WT
RL23A	60S ribosomal protein L23a	1.33E-02	APP23
WDR7	WD repeat-containing protein 7	1.45E-02	APP23
TAGL2	Transgelin-2	1.47E-02	APP23
DPYL2	Dihydropyrimidinase-related protein 2	1.48E-02	APP23
RAB14	Ras-related protein Rab-14	1.48E-02	APP23
HEXB	Beta-hexosaminidase subunit beta	1.49E-02	WT
SR140	U2 snRNP-associated SURP motif-containing protein	1.50E-02	APP23
CKAP5	Cytoskeleton-associated protein 5	1.52E-02	WT
GPM6A	Neuronal membrane glycoprotein M6-a	1.52E-02	APP23
ACACA	Acetyl-CoA carboxylase 1	1.52E-02	APP23
GHC2	Mitochondrial glutamate carrier 2	1.54E-02	APP23
MAON	NADP-dependent malic enzyme, mitochondrial	1.56E-02	APP23
TAGL3	Transgelin-3	1.56E-02	WT
MYPR	Myelin proteolipid protein	1.62E-02	APP23
SODM	Superoxide dismutase [Mn], mitochondrial	1.63E-02	APP23
MSRA	Mitochondrial peptide methionine sulfoxide reductase	1.64E-02	WT
ALG2	Alpha-1,3/1,6-mannosyltransferase ALG2	1.70E-02	WT

PP2BA	Serine/threonine-protein phosphatase 2B catalytic subunit alpha isoform	1.73E-02	APP23
ODPA	Pyruvate dehydrogenase E1 component subunit alpha, somatic form, mitochondrial	1.74E-02	APP23
HCN1	Potassium/sodium hyperpolarization-activated cyclic nucleotide-gated channel 1	1.77E-02	APP23
AP3B2	AP-3 complex subunit beta-2	1.82E-02	WT
PRAF3	PRA1 family protein 3	1.84E-02	APP23
SPCS	O-phosphoseryl-tRNA(Sec) selenium transferase	1.85E-02	WT
S12A5	Solute carrier family 12 member 5	1.90E-02	WT
NDRG1	Protein NDRG1	1.92E-02	WT/APP23
SPTN1	Spectrin alpha chain, non-erythrocytic 1	1.99E-02	APP23
APBB1	Amyloid-beta A4 precursor protein-binding family B member 1	2.04E-02	WT
OGT1	UDP-N-acetylglucosamine--peptide N-acetylglucosaminyltransferase 110 kDa subunit	2.04E-02	WT
SFXN3	Sideroflexin-3	2.04E-02	APP23
PHIPL	Phytanoyl-CoA hydroxylase-interacting protein-like	2.05E-02	APP23
NGEF	Ephexin-1	2.07E-02	WT
CCDC6	Coiled-coil domain-containing protein 6	2.10E-02	WT/APP23
TMX2	Thioredoxin-related transmembrane protein 2	2.13E-02	WT
HVM57	Ig heavy chain V region 6.96	2.15E-02	APP23
ARBK1	Beta-adrenergic receptor kinase 1	2.35E-02	WT
PLXB2	Plexin-B2	2.37E-02	APP23
PGP	Glycerol-3-phosphate phosphatase	2.37E-02	WT/APP23
NDUB2	NADH dehydrogenase [ubiquinone] 1 beta subcomplex subunit 2, mitochondrial	2.40E-02	WT/APP23
CRYM	Ketimine reductase mu-crystallin	2.41E-02	WT
ABR	Active breakpoint cluster region-related protein	2.46E-02	WT
HNRPU	Heterogeneous nuclear ribonucleoprotein U	2.48E-02	APP23
RAB21	Ras-related protein Rab-21	2.58E-02	WT
CNN3	Calponin-3	2.63E-02	WT/APP23
RHEB	GTP-binding protein Rheb	2.67E-02	WT
ITPR1	Inositol 1,4,5-trisphosphate receptor type 1	2.71E-02	WT
WASF1	Wiskott-Aldrich syndrome protein family member 1	2.83E-02	WT
MDHC	Malate dehydrogenase, cytoplasmic	2.84E-02	APP23
NC2A	Dr1-associated corepressor	2.87E-02	WT/APP23
NEUG	Neurogranin	2.88E-02	APP23
TIF1B	Transcription intermediary factor 1-beta	2.92E-02	APP23
COMD3	COMM domain-containing protein 3	2.92E-02	APP23
SC6A1	Sodium- and chloride-dependent GABA transporter 1	2.93E-02	WT/APP23
CHERP	Calcium homeostasis endoplasmic reticulum protein	2.97E-02	APP23
CYFP1	Cytoplasmic FMR1-interacting protein 1	2.97E-02	APP23
L1CAM	Neural cell adhesion molecule L1	3.17E-02	WT/APP23
ADHX	Alcohol dehydrogenase class-3	3.18E-02	WT
GLTP	Glycolipid transfer protein	3.21E-02	APP23
ACTG	Actin, cytoplasmic 2	3.22E-02	APP23
GRIA2	Glutamate receptor 2	3.29E-02	APP23
HVM54	Ig heavy chain V region 5-84	3.29E-02	APP23
ALDOC	Fructose-bisphosphate aldolase C	3.40E-02	APP23
ABCB8	ATP-binding cassette sub-family B member 8, mitochondrial	3.42E-02	APP23

ACADM	Medium-chain specific acyl-CoA dehydrogenase, mitochondrial	3.46E-02	WT
GARS	Glycine--tRNA ligase	3.48E-02	WT
RACK1	Receptor of activated protein C kinase 1	3.49E-02	WT
ACLY	ATP-citrate synthase	3.65E-02	WT
K0513	Uncharacterized protein KIAA0513	3.66E-02	APP23
SRP54	Signal recognition particle 54 kDa protein	3.67E-02	APP23
TMM65	Transmembrane protein 65	3.68E-02	APP23
HVM02	Ig heavy chain V region 93G7	3.70E-02	APP23
SAHH2	S-adenosylhomocysteine hydrolase-like protein 1	3.76E-02	APP23
MY18A	Unconventional myosin-XVIIIa	3.76E-02	WT
FUS	RNA-binding protein FUS	3.80E-02	WT
SCN9A	Sodium channel protein type 9 subunit alpha	3.82E-02	WT
SNAA	Alpha-soluble NSF attachment protein	3.85E-02	WT
PCBP1	Poly(rC)-binding protein 1	3.92E-02	APP23
ATP5J	ATP synthase-coupling factor 6, mitochondrial	3.96E-02	WT
E41L3	Band 4.1-like protein 3	3.99E-02	WT
RRAGC	Ras-related GTP-binding protein C	4.03E-02	WT
CMPK2	UMP-CMP kinase 2, mitochondrial	4.07E-02	WT
THUM1	THUMP domain-containing protein 1	4.09E-02	WT/APP23
SYT2	Synaptotagmin-2	4.16E-02	WT/APP23
COR1C	Coronin-1C	4.23E-02	APP23
ENOG	Gamma-enolase	4.25E-02	APP23
HS105	Heat shock protein 105 kDa	4.26E-02	WT
ODO1	2-oxoglutarate dehydrogenase, mitochondrial	4.28E-02	WT
QCR1	Cytochrome b-c1 complex subunit 1, mitochondrial	4.28E-02	APP23
SOGA3	Protein SOGA3	4.39E-02	WT
COX6C	Cytochrome c oxidase subunit 6C	4.41E-02	WT
HEMO	Hemopexin	4.45E-02	WT/APP23
MPC2	Mitochondrial pyruvate carrier 2	4.48E-02	APP23
BIEA	Biliverdin reductase A	4.49E-02	WT
ACOC	Cytoplasmic aconitate hydratase	4.60E-02	APP23
ALDR	Aldose reductase	4.64E-02	WT
AT1A1	Sodium/potassium-transporting ATPase subunit alpha-1	4.66E-02	APP23
PEF1	Peflin	4.67E-02	WT
IMPA1	Inositol monophosphatase 1	4.70E-02	APP23
LYNX1	Ly-6/neurotoxin-like protein 1	4.79E-02	WT
ELOB	Elongin-B	4.92E-02	WT
NSF1C	NSFL1 cofactor p47	5.00E-02	APP23
PRSR2	Proline and serine-rich protein 2	5.13E-02	WT
H33	Histone H3.3	5.17E-02	APP23
DNJA4	DnaJ homolog subfamily A member 4	5.23E-02	WT
ATPK	ATP synthase subunit f, mitochondrial	5.25E-02	WT
KV5AC	Ig kappa chain V-V region HP 93G7	5.33E-02	WT
U2AF1	Splicing factor U2AF 35 kDa subunit	5.37E-02	APP23
GBB2	Guanine nucleotide-binding protein G(I)/G(S)/G(T) subunit beta-2	5.40E-02	WT
PABP1	Polyadenylate-binding protein 1	5.45E-02	WT

ALBU	Serum albumin	5.45E-02	WT/APP23
GRM3	Metabotropic glutamate receptor 3	5.48E-02	APP23
AINX	Alpha-internexin	5.48E-02	APP23

The specificity of association with TG2 was evaluated by z-test analysis ($P \leq 0.05$) of n=5 animals per cohort, using the $TG2^{-/-}$ mice as background control (as outlined in Figure 3, A and B). Proteins are denoted by full name and UniProtKB protein entry name (ID), and they are listed according to the specificity of the interaction with TG2 (p-value). WT/APP23, TG2-associated proteins in both WT and APP23 brain (p-value listed is for WT); WT, TG2-associated proteins in WT brain; APP23, TG2-associated proteins in APP23 brain.
